# Speech characteristics yield important clues about motor function: Speech variability in individuals at clinical high-risk for psychosis

**DOI:** 10.1038/s41537-023-00382-9

**Published:** 2023-09-16

**Authors:** Kasia Hitczenko, Yael Segal, Joseph Keshet, Matthew Goldrick, Vijay A. Mittal

**Affiliations:** 1 Laboratoire de Sciences Cognitives et Psycholinguistique, Département d’Études Cognitives, ENS, EHESS, CNRS, PSL University, Paris, France; 2 Faculty of Electrical and Computer Engineering, Technion-Israel Institute of Technology, Haifa, Israel; 3 Department of Linguistics, Northwestern University, Evanston, IL USA; 4 Department of Psychology, Northwestern University, Evanston, IL USA; 5 Cognitive Science Program, Northwestern University, Evanston, IL USA; 6 Institute for Policy Research, Northwestern University, Evanston, IL USA; 7 Department of Psychiatry, Northwestern University, Evanston, IL USA; 8 Medical Social Sciences, Northwestern University, Chicago, IL USA; 9 Institute for Innovations in Developmental Sciences, Evanston/Chicago, IL USA

**Keywords:** Biomarkers, Psychosis, Human behaviour

## Abstract

Background and hypothesis: Motor abnormalities are predictive of psychosis onset in individuals at clinical high risk (CHR) for psychosis and are tied to its progression. We hypothesize that these motor abnormalities also disrupt their speech production (a highly complex motor behavior) and predict CHR individuals will produce more variable speech than healthy controls, and that this variability will relate to symptom severity, motor measures, and psychosis-risk calculator risk scores. Study design: We measure variability in speech production (variability in consonants, vowels, speech rate, and pausing/timing) in *N* = 58 CHR participants and *N* = 67 healthy controls. Three different tasks are used to elicit speech: diadochokinetic speech (rapidly-repeated syllables e.g., papapa…, pataka…), read speech, and spontaneously-generated speech. Study results: Individuals in the CHR group produced more variable consonants and exhibited greater speech rate variability than healthy controls in two of the three speech tasks (diadochokinetic and read speech). While there were no significant correlations between speech measures and remotely-obtained motor measures, symptom severity, or conversion risk scores, these comparisons may be under-powered (in part due to challenges of remote data collection during the COVID-19 pandemic). Conclusion: This study provides a thorough and theory-driven first look at how speech production is affected in this at-risk population and speaks to the promise and challenges facing this approach moving forward.

## Introduction

Individuals with psychosis exhibit motor abnormalities (e.g., tremors, rigidity, dyskinesia, soft-signs) and recent work has suggested that these behaviors may also represent sensitive prognostic indicators during the prodromal period^1–4^. In addition, motor signs can be objectively measured, in contrast to other symptom domains which are often subject to observer/rater bias^2,5^. However, motor assessments frequently require significant expertise, as well as time-intensive analyses and/or cumbersome instrumentation^2,6–9^. In this work, we explore one potential solution, examining the feasibility of using the physical properties of speech to measure motor abnormalities. Speech is a highly complex motor behavior, involving very fine-tuned movements that, when distorted in even subtle ways, can produce easily observable acoustic consequences (e.g., millimeter differences in placement/movement and millisecond differences in timing/coordination can substantially change the speech acoustics)^10^.

Because basal ganglia and cerebellar circuits modulate motor function and are also implicated in leading models of psychosis^11–13^, there is good reason to believe that motor signs may be an early and sensitive biomarker^4^. Indeed, of the identified early vulnerability markers seen in children that develop adult psychosis, motor abnormalities may be the most common^14^. For example, a myriad of motor behavior domains have been demonstrated to predict infants/children that ultimately develop adult schizophrenia including: delays in achieving motor milestones^15^, neuromotor deficits and involuntary movements^16^, and neurological soft signs^17^. One study comparing childhood video tapes of schizophrenia patients with childhood videos of their healthy siblings as well as healthy community controls, found that the pre-schizophrenia children showed a higher rate of motor abnormalities and delays^18^. In a similar study, Schiffman and colleagues^19^ examined video-taped social interactions of 11–13 year old children who later developed schizophrenia and observed that a high occurrence of movement abnormalities distinguished the pre-schizophrenia children from matched controls. In adolescence, neuromaturational factors and environmental stressors can exacerbate underlying vulnerabilities in the motor and dopamine system^20^, leading to other outward manifestations in this age group, including spontaneous dyskinesias (i.e., spontaneous jerking and irregular ballistic movements)^21^. Indeed, among high-risk groups (i.e., those showing a low level of symptoms) these particular motor behaviors increase in frequency and severity as a function of development and increased disease burden, are associated with increased attenuated positive symptoms^22,23^ and strongly predict conversion to psychosis^24–26^. As not all at-risk individuals go on to develop a psychotic disorder, this is highly relevant^27^. Irrespective of medication (i.e., the motor abnormalities are present in neuroleptic naïve samples), these spontaneous jerking movements in the head, face, lips, and torso can continue to emerge during the adolescent prodromal period, until onset, when they remain a key clinical feature of the illness^28^. At least one cross-sectional study suggests that with advanced age, all patients with schizophrenia will eventually develop these behaviors^29^. We point readers to recent review articles for more discussion of motor function in the prodromal syndrome^2,27,30–34^.

Previous work has examined speech production in schizophrenia/psychosis^35–44^. This work has been promising, but results are mixed. A recent meta-analysis found three speech measures (speech rate, pause duration, and proportion spoken time) differentiated clinical and control groups (individuals with schizophrenia had slower speech rates, longer pauses, and lower speaking proportions), but only one (pause duration) showed a large effect^43^. In addition, the meta-analysis reported differences in results depending on the speech task used, generally finding that more cognitively or socially demanding tasks (e.g., free speech or dialogues) resulted in larger effects. However, this prior work has generally not examined individuals at clinical high-risk (CHR), nor has it focused on motor abnormalities. It also has largely pursued a data-driven approach. Our work pursues a hypothesis-driven approach, studying acoustic measures that are predicted to be disrupted by motor abnormalities in speech produced by individuals at clinical-high-risk.

We hypothesize that disruptions to motor control will impact control over vocal articulators (e.g., tongue, lips), leading to more variable speech in CHR participants when compared to healthy controls (HC), analogous to what has been observed in speech disorders^45–47^. Furthermore, if these speech measures reflect motor abnormalities, we would expect that increased variability in speech productions should relate to other measures of motor abnormalities (e.g., finger-tapping, as a test of convergent validity), worse symptom severity (as a test of clinical validity), and higher risk of conversion to psychosis (as a test of predictive validity). To systematically examine the conditions under which motor difficulties are observed, we elicit speech in highly-controlled samples that are specifically designed to measure motor difficulties (diadochokinetic speech), read speech, as well as more free-form, naturalistic speech which closely resembles everyday speech.

## Results

We present results by speech task. We focused on acoustic speech measures that have been extremely well-studied and can be reliably measured automatically (which allows us to study greater quantities of speech). The main text focuses on (i) variability in the voice-onset-time of voiceless (in English: p,t,k) and voiced (in English, b,d,g) stop consonants, i.e., the primary acoustic measure of stop consonants, defined as the duration between the release of the consonant and the onset of the following vowel, (ii) variability in vowel durations, and (iii) variability in speech rates. We discuss the remaining speech measures we studied (including variability in vowel formants and variability in pausing/timing)^48–53^ in Table 1 and Supplementary Materials 1.3.

**Table 1:** Summary of the studied speech measures by speech task.

		Diadochokinetic-AMR	Diadochokinetic-SMR	Read	Spontaneous
*Consonant production measures*					
CoV of voiceless stop VOTs	CHR	*0.31 (0.09)*	**0.39 (0.1)**	**0.43 (0.1)**	0.4 (0.09)
	HC	*0.27 (0.1)*	**0.35 (0.09)**	**0.39 (0.07)**	0.4 (0.07)
	*p*	*0.05*	**0.02**	**0.03**	0.73
CoV of voiced stop VOTs	CHR			0.7 (0.26)	0.66 (0.26)
	HC			0.71 (0.26)	0.58 (0.31)
	*p*			0.67	0.18
*Speech rate measures*					
CoV of speech rate	CHR	**0.07 (0.04)**	**0.1 (0.05)**	**0.2 (0.05)**	0.43 (0.45)
	HC	**0.05 (0.04)**	**0.08 (0.04)**	**0.18 (0.06)**	0.37 (0.32)
	*p*	**<0.01**	**0.01**	**0.04**	0.52
*Vowel production measures*					
CoV of vowel durations	CHR	0.2 (0.09)	0.43 (0.12)	0.56 (0.05)	0.74 (0.11)
	HC	0.17 (0.09)	0.4 (0.15)	0.56 (0.06)	0.73 (0.09)
	*p*	0.13	0.18	0.42	0.72
Formant dispersion 20%	CHR	**158.63 (86.4)**	*211.7 (85.72)*	341.76 (53.41)	357.52 (48.52)
	HC	**128.85 (64.57)**	*185.02 (75.8)*	332.5 (48.44)	362.32 (58.29)
	*p*	**0.03**	*0.08*	0.33	0.77
Change in formant dispersion 20–50%	CHR	14.59 (21.02)	20.33 (20.06)	−22.07 (14.82)	−4.5 (16.36)
	HC	10.68 (20.01)	19.41 (19.17)	−21.99 (15.2)	−2.06 (17.79)
	*p*	0.33	0.81	0.98	0.48
Overlap between vowel categories	CHR			0.8 (5.91)	0 (0)
	HC			0 (0)	0 (0)
	*p*			0.64	0.45
*Timing/pausing measures*					
CoV of syllable durations	CHR	0.21 (0.09)	0.39 (0.1)		
	HC	0.17 (0.08)	0.36 (0.13)		
	*p*	0.11	0.1		
CoV of intersyllable durations	CHR	*0.42 (0.25)*	0.9 (0.27)		
	HC	*0.34 (0.18)*	0.82 (0.23)		
	*p*	*0.05*	0.17		
Number of pauses	CHR			0.1 (0.03)	0.15 (0.05)
	HC			0.09 (0.03)	0.14 (0.07)
	*p*			0.13	0.4

For each speech measure/speech task combination, the table provides descriptive statistics [mean (standard deviation)] by group (CHR vs. HC), as well the *p*-value corresponding to the CHR vs. HC group difference test. Blank cells indicate that the speech measure in question was not calculated for the speech task in question. Measures are bolded if they show a significant CHR vs. HC group difference and italicized if just above significance. *CoV* coefficient of variation, *VOT* voice-onset-time.

N.B.: One CHR participant was identified as clinical high-risk in-remission and another participant had a 7-months’ gap between their clinical interview and speech tasks. Supplementary Materials 2.10 includes analyses without these two participants; the results are qualitatively similar to the analyses of the full dataset reported below.

### Diadochokinetic speech tasks

Participants first completed a diadochokinetic speech task, in which they produced particular syllable types as quickly and as accurately as possible^54,55^. This task consisted of two trial types that we analyze separately: Alternating Motion Rate (AMR) trials, in which participants repeated a single target syllable 15 times (e.g., pa-pa-pa…, ta-ta-ta…, ka-ka-ka…) and Sequential Motion Rate (SMR) trials, in which they repeated sequences of three syllables 10 times (e.g., pa-ta-ka…, ka-ta-pa…).

**Fig. 1:**
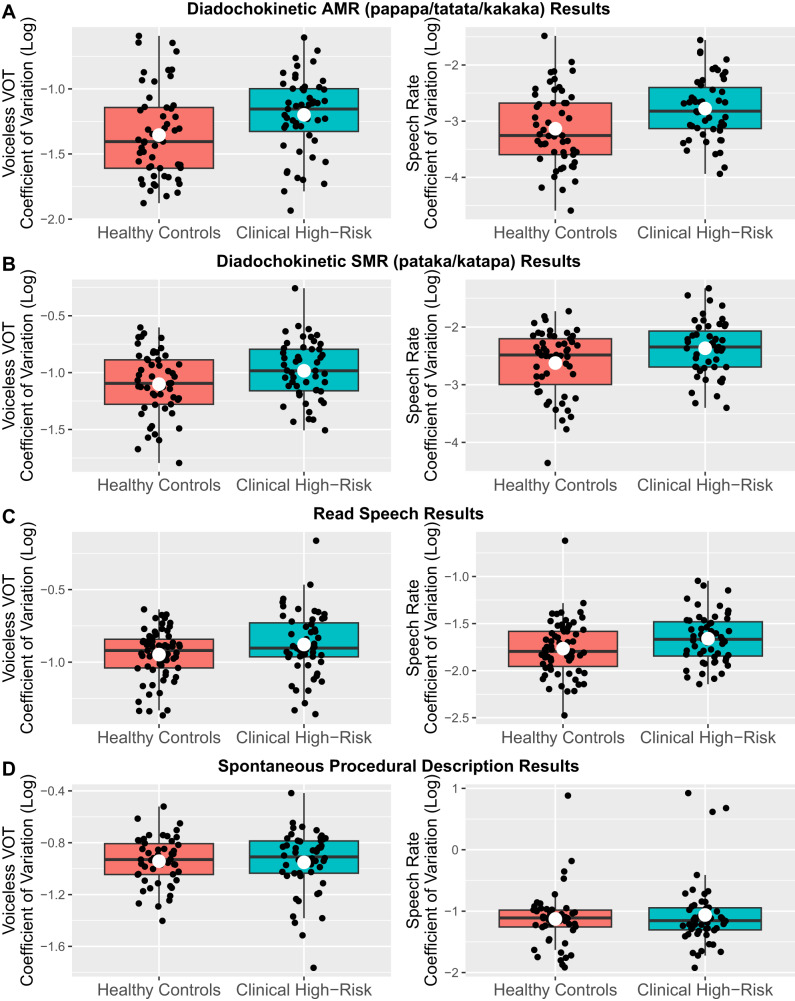
CHR vs. HC group differences in consonant and speech rate variability across the four studied speech tasks. CHR individuals produce more variable consonants (left plot in each row) and speech rates (right plot in each row) compared to controls in **A** diadochokinetic-AMR, **B** diadochokinetic-SMR, and **C** read speech, but not **D** spontaneous speech. Each black dot is one participant; the white dot is the average across participants.

Out of the seven speech measures we studied (Table 1), we found evidence that CHR individuals produced more variable voiceless stop consonant voice-onset-times than HC—near significantly in AMR trials ($\beta$ = 0.09, s.e. = 0.05, *t* = 1.95, *p* = 0.054; Fig. 1A) and significantly in SMR trials ($\beta$ = 0.12, s.e. = 0.05, *t* = 2.45, *p* = 0.016; Fig. 1B). CHR individuals also produced more variable speech rates than HC in both AMR and SMR trials (AMR: $\beta$ = 0.36, s.e. = 0.12, *t* = 2.98, *p* = 0.004; SMR: $\beta$ = 0.26, s.e. = 0.1, *t* = 2.52, *p* = 0.013; with one exception, all other speech measures showed no significant effects). However, these two measures generally did not correlate with SIPS scores, finger-tapping, or risk scores (results in Table 2 and Supplementary Materials 2.1).

**Table 2:** Correlations between speech measures that show CHR vs. HC group differences and non-speech motor/clinical/risk validation measures. Note that the spontaneous speech task is not included as we did not observe significant group differences in that task.

	Diadochokinetic-AMR		Diadochokinetic-SMR		Read Speech	
	Voiceless VOT CoV	Speech rate CoV	Voiceless VOT CoV	Speech rate CoV	Voiceless VOT CoV	Speech rate CoV
SIPS positive total	*r* = 0.1 (*p* = 0.79)	*r* = 0.19 (*p* = 0.19)	*r* = 0.08 (*p* = 0.84)	*r* = 0.07 (*p* = 0.62)	*r* = 0.28 (*p* = 0.12)	*r* = 0.07 (*p* = 0.62)
SIPS negative total	*r* = 0.23 (*p* = 0.28)	*r* = 0.07 (*p* = 0.64)	*r* = 0.22 (*p* = 0.32)	*r* = 0.18 (*p* = 0.21)	*r* = 0.17 (*p* = 0.48)	*r* = 0.05 (*p* = 0.7)
SIPS disorganized total	*r* = 0.07 (*p* = 0.9)	*r* = 0.26 (*p* = 0.08)	*r* = 0.28 (*p* = 0.16)	*r* = 0.13 (*p* = 0.37)	*r* = 0.2 (*p* = 0.38)	*r* = 0.02 (*p* = 0.89)
SIPS G3 (motor)	*r* = 0.15 (*p* = 0.62)	*r* = 0.11 (*p* = 0.48)	*r* = 0.2 (*p* = 0.39)	*r* = 0.06 (*p* = 0.68)	*r* = 0.26 (*p* = 0.2)	*r* = 0.11 (*p* = 0.45)
Finger-tapping CoV (dominant hand)	*r* = 0.06 (*p* = 0.92)	*r* = 0.08 (*p* = 0.6)	*r* = 0.08 (*p* = 0.87)	*r* = 0.09 (*p* = 0.54)	*r* = 0.17 (*p* = 0.48)	*r* = 0.11 (*p* = 0.47)
Finger-tapping CoV (non-dominant hand)	*r* = 0.15 (*p* = 0.62)	*r* = 0.29 (*p* = 0.05)	*r* = 0.05 (*p* = 0.96)	*r* = 0.1 (*p* = 0.51)	*r* = 0.18 (*p* = 0.49)	*r* = 0.39 (*p* = 0.01)
SIPS-RC risk score	*r* = 0.4 (*p* = 0.02)	*r* = 0.07 (*p* = 0.65)	*r* = 0.32 (*p* = 0.1)	*r* = 0.09 (*p* = 0.53)	*r* = 0.13 (*p* = 0.66)	*r* = 0.1 (*p* = 0.51)

### Read speech

**Fig. 2:**
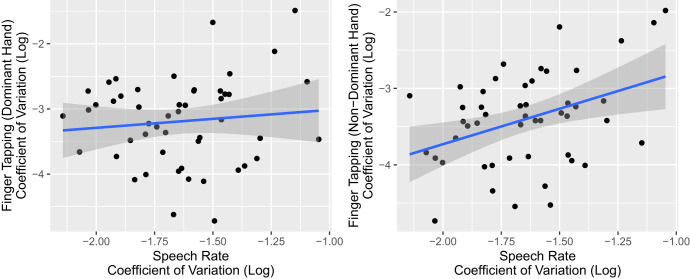
Correlation between variability in speech rate and variability in finger-tapping rate in the read speech task. We observe a significant positive relationship between variability in speech rate and variability in finger-tapping rates in the non-dominant hand (left plot), but not the dominant hand (right plot). Each point represents one participant; the line of best-fit is shown, with shaded regions showing standard errors of the regression fit.

Participants then read a standardized passage aloud at a comfortable pace (full text in Supplementary Materials 1.1). As in the diadochokinetic speech task, we found that CHR individuals produced more variable voiceless stop consonant voice-onset-times ($\beta$ = 0.08, s.e. = 0.04, *t* = 2.23, *p* = 0.028) and speech rates ($\beta$ = 0.11, s.e. = 0.05, *t* = 2.1, *p* = 0.038) than HC (Fig. 1C; all other speech measures showed no significant effects). Variation in speech rate (but not consonant voice-onset-time) was significantly positively correlated with another motor measure, variability in finger-tapping rate in the non-dominant hand ($\beta$ = 0.93, s.e. = 0.32, *t* = 2.86, *p* = 0.006), but not in the dominant hand ($\beta$ = 0.28, s.e. = 0.37, *t* = 0.74, *p* = 0.465; Fig. 2). However, these measures did not correlate with clinical or risk measures (results in Table 2 and Supplementary Materials 2.2).

### Spontaneous speech

Finally, we elicited spontaneous speech, by asking participants to describe how to make a peanut butter and jelly sandwich for ~2 min. In contrast to the diadochokinetic and read speech samples, we found that none of the speech measures differed by group status in spontaneous speech (Fig. 1D), including the two measures impacted in the previous tasks: variability in voiceless consonant voice-onset-time ($\beta$ = −0.01, s.e. = 0.04, *t* = −0.34, *p* = 0.732) and variability in speech rate ($\beta$ = 0.06, s.e. = 0.1, *t* = 0.64, *p* = 0.524). Because none of the acoustic speech tasks showed significant results (Supplementary Materials 2.3), we did not test for associations with non-speech motor/clinical/risk measures.

### In-person vs. remote results

**Fig. 3:**
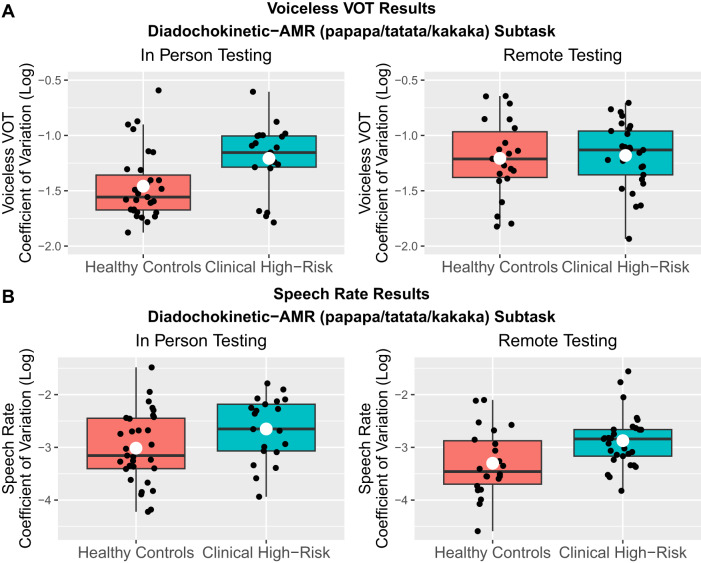
Comparison of results between in-person vs. remote testing conditions. **A** Group differences in consonant variability only emerged in in-person (left plot), not remote (right plot) testing conditions. **B** Group differences in speech rate variability were qualitatively similar across the in-person and remote subgroups (example plots from diadochokinetic-AMR speech). Each black dot represents one participant; the white dot represents the average value across participants.

Because data collection occurred between 2019–2022, our study had to be adapted to the remote format partway through due to the COVID-19 pandemic (see Methods for details). In post-hoc analyses, we tested whether results differed between participants tested in-person (*N* = 70) vs. remotely (*N* = 52), focusing on the measures and tasks that showed group differences in our primary analyses (Fig. 3 and S20–S21). Full results are presented in Supplementary Materials 2.7, but we generally observed smaller group differences in consonant (voice-onset-time) variability in the remote subgroup relative to the in-person group. This seemed to be driven by greater variability in the remotely-recorded control group relative to the in-person control group. For speech rate, however, the in-person and remote subgroups showed qualitatively similar patterns, except in the diadochokinetic-SMR subtask, where we again observed a reduction in CHR vs. HC group differences when tested remotely.

### Unpacking why we did not see a relationship with clinical/motor symptoms

Contrary to our predictions, we found that the speech measures that showed CHR vs. HC group differences did not correlate with motor, clinical, or risk measures. We ran several additional exploratory analyses in an attempt to unpack this surprising finding.

Past work has shown that some linguistic measures are highly correlated with sociodemographic factors^56–58^. To verify this was not the case for the speech measures we studied, we ran regressions predicting demographic factors (age, sex, race, native language) from the speech measures that significantly differed between the high-risk and healthy control groups. We found no significant relationships, suggesting that the observed group differences were not accounted for by demographic factors (see Supplementary Materials 2.8).

We then tested whether the non-speech (motor/clinical/risk) measures correlated with one another as we would expect based on previous work^7,59^. They did not. In our sample, individuals with greater motor abnormalities (measured by finger-tapping) did not have worse overall symptoms or higher risk of conversion scores (see Supplementary Materials 2.9 for results). This suggests we did not have sufficient power to detect motor abnormalities. Indeed, past work has found a $r$ = 0.37 correlation between finger tapping speed and total negative symptoms in the CHR group^7^. Assuming the effect size is similar for finger-tapping variability and speech variability, which we measure here, a post-hoc power analysis suggests that, in the best case scenario (i.e., without a midway shift to remote testing), we would need a sample size of *N* = 56 (with $\alpha$ = 0.05 and $\beta$ = 0.9) to detect this effect size, whereas our analysis had sample sizes ranging from *N* = 47–51^60^.

## Discussion

We find evidence that individuals at clinical high-risk for psychosis produce more variable speech - in particular, more variable consonant voice-onset-times and speech rates—than healthy controls in two of the three speech types we study. However, contrary to predictions, we found that increased speech variability did not correlate with non-speech motor measures, symptom severity, or conversion risk scores. Follow-up analyses suggest that these comparisons may have been underpowered and, in particular, affected by a midway shift from in-person to remote testing. This theory-driven analysis provides a thorough first look at how speech production is affected in the CHR population and speaks to the promise and challenges facing this approach to measuring motor symptoms.

### Not all aspects of speech are affected and not in all contexts

Our findings converge with Parola et al.’s^43^ meta-analysis, which found that other aspects of speech rate were one of the three strongest acoustic factors differentiating groups (n.b. they did not study voice-onset-time or speech rate variability). This stands in notable contrast to past findings in the field, which generally showed mixed results across studies (i.e., the particular speech measures that differed between groups differed depending on the study and speech samples; see discussion in Hitczenko et al.^61^). We believe this consistency across speech tasks and convergence with past studies reflects the benefits of adopting a theory-driven approach when studying highly variable speech signals.

That being said, most of the speech measures did not show group differences. In particular, we failed to find effects of variability of voiced consonant voice-onset-times (b,d,g), which likely reflects motor control demands. Specifically, English voiceless consonants involve more motor coordinating/timing than voiced consonants, as the vocal folds need to be suppressed for a specific amount of time^62–67^. We also failed to find the expected effect for vowels. This is less expected, but one possibility is that it may reflect the more precise articulatory and timing requirements for stop consonants, which are overall much shorter than vowels.

In addition to variable results across measures, group differences only appeared in some of the speech tasks: diadochokinetic-AMR, diadochokinetic-SMR, and read speech, but not spontaneous speech. This may reflect the degree to which different tasks present challenges to speech articulation. Diadochokinetic speech involves unnatural rapid repetition of syllables, while the passage participants read includes many low frequency words (e.g., Aristotle, bow, refraction). Indeed, qualitatively, participants often remarked on the difficult aspects of these tasks, or produced disfluent speech. These sorts of targeted, more challenging speech tasks may be necessary for detecting the impact of motor disruptions on speech articulation. Another possibility is that this simply reflects statistical power; the spontaneous task was shorter, and the content was more variable, which may have washed out subtle differences between groups.

In addition, there was some evidence of differences between in-person and remote participants. Healthy control individuals generally had more variable speech measures when tested remotely vs. in-person, reducing our ability to detect group differences. There has been substantial recent interest in developing remote options for all manner of clinical assessment (e.g., to reach individuals who are medically underserved^68^), but this result reveals that these approaches need to be carefully developed and validated. In the case of speech measures specifically, our results likely reflect issues in an analysis pipeline that was developed to analyze speech recorded in controlled laboratory conditions. More broadly, expanding access to such assessments requires developing analysis methods that are robust to variation in testing and recording conditions.

Finally, when group differences did emerge, they were subtle. In particular, the distributions over speech measures between the CHR and HC subgroups overlapped substantially. Predicting group membership from individual speech measures yielded categorization accuracy rates between 60-65%, which is typically considered inadequate (see Supplementary Materials 2.6 for categorization analyses)^69,70^. It is important to stress that these measures are not diagnostic on their own (after all, speech is affected by a large number of interacting factors, only one of which is motor abilities). Nonetheless, the fact that we observe group differences and above-chance categorization rates supports the notion that theoretically-motivated speech measures, in conjunction with other sources of information, could be useful for diagnostics down the line, and future work should continue to study this possibility.

### Speech measures did not correlate with motor, clinical, or conversion risk variables

While the observed group differences provide converging support that speech/motor symptoms are observed very early in the progression of psychosis, the biggest challenge facing these speech measures is that they mostly did not correlate with clinical/motor/risk variables. This could reflect insufficient power. We had non-speech motor symptoms for ~50 CHR participants, which would only let us detect effect sizes of ~0.39 or higher ($\alpha$ = 0.05; $\beta$ = 0.9). Exploratory analyses studying the relationship between motor and clinical measures in our sample (Supplementary Materials 2.9) suggest that our particular sample and measures may have been insufficient to detect the typical clinical-high-risk motor profile, which would also weaken our ability to detect speech-motor relationships. Relatedly, the clinical-high-risk participants in our sample all had a relatively low risk of conversion (i.e., risk scores of 10.1% or lower), so there may not have been enough variability in clinical status in our sample to detect significant effects between speech measures and risk/symptom severity scores. Finally, we had to adapt our data collection procedure partway through to adhere to pandemic-related restrictions, including shortening the finger-tapping task and collecting speech samples remotely (participants were mailed audio recorders to their homes). While these changes were unavoidable, they reduced our power (e.g., by reducing the number of finger-tapping observations) and may have affected reliability, by introducing noise into our measures. We provided extensive guidelines, but ultimately had limited control over the participants’ environment (e.g., how noisy it was) and equipment (e.g., keyboard). Beyond practical task differences, the pandemic also may have had a substantial effect on individuals’ mental health, further increasing variability^71,72^.

Nonetheless, even though the speech measures did not correlate with clinical/motor variables, the fact that they differed by clinical status (CHR vs. HC), which is assigned based on clinical interview, means that, on some level, these measures must be related to symptomatology. In addition, the speech differences we observe could reflect motor abnormalities that are not captured by previously-developed measures (e.g., finger-tapping). In this case, we would not expect to see a correlation between the speech measures and previously-developed measures, but the speech measures would nonetheless be clinically informative. In sum, these differences are worthy of further investigation to understand what these speech measures reflect and how they can help researchers/clinicians.

### Recommendations for future approaches

Based on our results, future studies should prioritize difficult speech tasks that specifically target the speech feature of interest (e.g., diadochokinetic speech involving both voiceless/voiced consonants and a variety of vowels; sustained phonation tasks). An additional benefit of the more targeted measures is that they are easily transferable across other languages (many languages have the diadochokinetic speech syllables), which will be critical for establishing the validity and generalizability of these measures across populations^73^.

It would also be informative to systematically vary the phonetic (and other types of) complexity of the speech stimuli used (as in Kuruvilla-Dugdale et al.^74^), in order to systematically test whether more difficult speech stimuli better reveal the subtle differences in motor performance between clinical-high-risk and healthy participants, and are more sensitive to clinical severity/risk. Future studies should also study other motor measures that have been shown to capture motor/cerebellar abnormalities in early psychosis (e.g., pursuit rotor procedural learning tasks^75^, in which participants track a moving target with a computer mouse, or postural sway tasks^9,76^, in which participants’ balance is evaluated in various standing conditions). Because many existing motor tasks are difficult to adapt to remote testing, the COVID-19 pandemic limited the motor measures we could collect from our participants, but these tasks tap into distinct components of motor control (timing, motor learning, coordination, etc.), and determining which (if any) of them correlate with the speech measures we study will be important.

Finally, the clinical-high-risk group is heterogeneous and future work should identify and study well-motivated subgroups^26,77,78^. This is important because speech is affected by motor abilities, but also many other factors. For example, past work has often studied speech as a window into negative symptoms^43^. Even within the motor domain, it is possible that several motor networks may be impacted in this population (e.g., some individuals may show increased motor variability, while others may exhibit catatonia, or a reduction in movement variability/increase in rigidity)^4,59,79^, and that numerous distinct motor signs may be present in the same individuals^26^. While the motor deficits we focus on here should result in *more* speech variability, researchers adopting other focuses may predict that individuals will exhibit *less* speech variability. Competing effects of this sort could obscure a clear relationship between speech and symptoms. To address this issue, future work could collect a larger clinical-high-risk sample and identify subgroups (e.g., one that primarily shows negative symptoms, one that primarily shows increased motor variability, one that shows increased motor rigidity) and test whether they show different speech profiles in accordance with their different symptom profiles.

Overall, however, while many questions remain, the present work provides a solid foundation for future work investigating the insights that speech production can provide for understanding the mechanisms impacted in individuals at clinical-high-risk for psychosis.

## Methods

### Participants

*N* = 122 participants (*N* = 56 CHR; *N* = 66 HC) provided speech data, though not everybody provided data for all three tasks. The data of two CHR participants were excluded: one dropped out of the study and the other was later determined to have been erroneously classified as high-risk. This left *N* = 104 (*N* = 51 CHR; *N* = 53 HC) diadochokinetic speech samples, *N* = 120 (*N* = 55 CHR; *N* = 65 HC) read speech samples, and *N* = 100 (*N* = 50 CHR; *N* = 50 HC) spontaneous speech samples. The Structured Interview for Prodromal Syndromes (SIPS) was used to determine the clinical status of each participant (CHR vs. HC)^80^. See Table 3 for participant demographics.

**Table 3:** Summary of participant demographic information.

	Clinical high-risk (CHR)	Healthy controls (HC)
*N*	56 (In-Person: 26; Remote: 30)	66 (In-Person: 44; Remote: 22)
Sex (% Female)	60.7% (In-Person: 50%; Remote: 70%)	62.1% (In-Person: 59.1%; Remote: 68.2%)
Age (SD)	21.8 (2.8) (In-Person: 21.5 (2.4); Remote: 22.1 (3.1))	21.7 (3.2) (In-Person: 21.2 (3); Remote: 22.7 (3.5))
Race	44.6% White; 19.6% Black; 17.9% Asian; 8.9% Central/South American; 1.8% Native Hawaiian or Pacific Islander; 7.2% Multiracial (1.8% First Nations & White; 1.8% Black & White; 3.6% Asian & White) (In-Person: 38.5% White; 30.8% Black; 19.2% Asian; 11.5% Central/South American; Remote: 50% White; 10% Black; 16.7% Asian; 6.7% Central/South American; 3.3% Native Hawaiian or Pacific Islander; 13.2% Multiracial (3.3% First Nations & White; 3.3% Black & White; 6.7% Asian & White))	45.5% White; 9.1% Black; 28.8% Asian; 3% First Nations; 13.6% Multiracial (1.5% First Nations & White; 1.5% Black & White; 3% Asian & White; 7.6% not reported) (In-Person: 50% White; 13.6% Black; 25% Asian; 2.3% First Nations; 9.1% Multiracial (not reported); Remote: 36.4% White; 36.4% Asian; 4.5% First Nations; 22.7% Multiracial (4.5% First Nations & White; 4.5% Black & White; 9.1% Asian & White; 4.5% not reported))
Ethnicity	26.8% Hispanic; 73.2% Not Hispanic (In-Person: 23.1% Hispanic; Remote: 30% Hispanic)	10.6% Hispanic; 89.4% Not Hispanic (In-Person: 11.4% Hispanic; Remote: 9.1% Hispanic)
First Language	62.5% English; 17.9% Other; 19.6% Not reported (In-Person: 73.1% English; 26.9% Other; Remote: 53.3% English; 10% Other; 36.7% Not reported	80.3% English; 18.2% Other; 1.5% Not reported (In-Person: 81.8% English; 15.9% Other; 2.3% Not reported; Remote: 77.3% English; 22.7% Other)

### Speech tasks

Working one-on-one with an experimenter, participants provided three speech samples, recorded via a Zoom H2n portable audio recorder (44.1 kHz sample rate; 16-bit recording; X/Y recording configuration; no compression/limiting or low-cut filtering was used). Participants were seated 16 inches from the recorder and worked with the experimenter to ensure proper audio/gain levels prior to recording.

#### Diadochokinetic speech task

Participants first completed a diadochokinetic speech task, commonly-used to examine speech motor abilities, in which they were asked to produce particular syllable types as quickly and accurately as possible^54,55^. They first produced 12 Alternating Motion Rate (AMR) trials, repeating a target syllable 15 times (two trials each of: pa-pa-pa…, ta-ta-ta…, ka-ka-ka…, ba-ba-ba…, da-da-da…, ga-ga-ga…). They then produced 20 Sequential Motion Rate (SMR) trials, producing sequences of three syllables 10 times each per trial (10 trials each of pa-ta-ka… and ka-ta-pa…).

#### Read speech task: Rainbow passage

Participants then read aloud the Rainbow Passage at a comfortable pace (passage in Supplementary Materials 1.1)^81^. The passage is commonly-used for eliciting read speech, as it is phonetically balanced (covering all English speech sounds) and emotionally neutral. The Rainbow Passage has quite a few low-frequency words (e.g., “Aristotle”, “refraction”), so it is relatively difficult to read. This speech task allows us to precisely control the speech content, while eliciting a more naturalistic speaking style than diadochokinetic speech.

#### Spontaneous procedural description task: Peanut butter and jelly

Finally, we elicited spontaneous speech, by asking participants to describe how to make a peanut butter and jelly sandwich for ~2 min. Unlike the other tasks, the speech content differed between participants (though many words overlapped: e.g., peanut, butter, knife). Such procedural description tasks are less emotionally and cognitively demanding than personal narratives while still generating a large volume of speech^82^.

### Speech measures

At a high-level, for each participant, for each speech sample, we estimated how variable (i) their consonant productions, (ii) their vowel productions, (iii) their speech rates, and (iv) their pausing/timing were using semi-automated methods^83–86^. Semi-automated methods greatly increase the amount of speech we can study, as extracting speech measures by-hand is extremely time-consuming, and ensure that our measurements are consistent and replicable (analysis code is available at github.com/khitczenko/chr_speech; the National Institute of Mental Health Data Archive provides de-identified clinical, risk, and demographic information). As a result, we focused on speech measures that have been extremely well-studied and can be reliably measured automatically. The main text focuses on (i) variability in the voice-onset-time of stop consonants (in English: p,t,k,b,d,g), or the time that elapses between the release of the consonant and the onset of the following vowel, (ii) variability in the duration of vowels, and (iii) variability in speech rates (calculated at the syllable level). We discuss the remaining speech measures we studied^48–53^ in Table 1 and Supplementary Materials 1.3.

For the diadochokinetic speech samples, we used DDKtor^86^ (https://github.com/MLSpeech/DDKtor) - a deep neural network model specifically trained to match human annotations of diadochokinetic speech - to automatically obtain the onset and offset (and thus, duration) of each stop consonant, vowel, and syllable given hand-selected windows of analysis which corresponded to the individual diadochokinetic trials. For the read and spontaneous speech, we first used the Montreal Forced Aligner^85^ (https://github.com/MontrealCorpusTools/Montreal-Forced-Aligner) to automatically align a transcript we created for each speech sample to its audio. The Montreal Forced Aligner uses a pronunciation dictionary and a trained acoustic model (specifically, a GMM-HMM model triphone model trained from MFCC features) to provide the onset and offset (and thus, duration) of each vowel, consonant, and syllable produced. We then applied AutoVOT^87,88^—a discriminative learning algorithm trained to match human voice-onset-time measurements - to the aligner output to obtain even more reliable onsets and offsets for the stop consonant voice-onset-times. Finally, we used the FastTrack software^84^ (https://github.com/santiagobarreda/FastTrack) to automatically obtain measurements of the first and second formant for each vowel that was output either from DDKtor (for diadochokinetic speech) or from the Montreal Forced Aligner (for read and spontaneous speech). FastTrack uses linear predictive coding to systematically identify candidate formant analyses for each vowel, from which it selects one winning analysis based on the smoothness of the predicted formant contours. We followed all recommendations provided by the creators of these tools (see Supplementary Materials 1.2 for full details).

These automated tools have been evaluated in the context of previous work and are highly reliable. The DDKtor software matches human annotations of diadochokinetic segment duration with correlations of *r* = 0.85-0.90 and matches human annotation of diadochokinetic speech rate with correlations of *r* = 0.94–0.97^86^. The Montreal Forced Aligner has an average phone boundary error of ~20 ms, across both isolated word productions and conversational speech, comparable to human interrater reliability^85^. For the AutoVOT software, ~90% of its predicted voice-onset-times are within 10–15 ms of gold-standard human annotation, again paralleling interrater reliability rates^87,88^. Finally, FastTrack has an average error of ~20 Hz and 98.9% of vowels have errors of less than 5% of the human-annotated value^84^. Overall, these tools perform comparably to human annotators and, when applied to our speech samples specifically, result in valid measurements that match expected average values (Supplementary Materials 1.4).

We measure variability using coefficients of variation, which control for potential differences in means, calculated as follows^89^:

$${{\rm{Coefficient}}\, {\rm{of}}\, {\rm{Variation}}}=\frac{{\rm{Standard}}\, {\rm{Deviation}}}{\rm{Mean}}$$

All measures are log-transformed in the analyses (as they tend to be skewed right, due to lower bounds at 0).

#### Variability in consonant duration

We focused on syllable-initial stop consonants (in English: p, t, k, b, d, g) that precede vowels (e.g., the bolded sounds in “**p**asser**b**y”, “**p**ulp”, “**p**eanut”, but not the “p” in “prism”), as they have easily-measurable acoustics^45,90^. In the diadochokinetic speech tasks, we restricted our analysis even further to only include voiceless stop consonants (in English: p, t, k), as the automated tool we use for this task has only been validated for this subset. We used the voice-onset-time duration of each consonant to calculate: (i) the coefficient of variation over voiceless stop (p,t,k) consonants (all speech samples) and (ii) the coefficient of variation over voiced stop (b,d,g) consonants (read and spontaneous speech only).

#### Variability in vowel duration

We focused on vowels that bear primary stress (e.g., only the bolded sounds: “**e**lement”, “aw**a**ken”, “an**a**lysis”) as they have easily-measurable acoustics. We used each relevant vowel’s duration to calculate the coefficient of variation across vowel tokens in each speech sample.

#### Variability in speech rate

Speech rate was calculated as the number of syllables participants produced per second. For diadochokinetic speech, we calculated the speech rate of each individual trial and calculated the coefficient of variation across all trials produced. For read and spontaneous speech, we calculated the speech rate of delimited phrases, defined as any spoken interval between silences of at least 150 ms^91^. We then calculated the coefficient of variation across all produced phrases.

### Non-speech validation measures

We used symptom severity measures, non-speech motor measures, and risk measures to establish the clinical, convergent, and predictive validity of the speech measures.

#### Clinical utility: Symptomatology

We assessed symptom severity with SIPS scores^80^. We focused particularly on the positive symptoms, negative symptoms, and disorganized symptoms totals to study how broadly clinically useful the speech measures are. In addition, we looked at the individual item G3 (“Motor Difficulties”—i.e., have you noticed any clumsiness, awkwardness, or lack of coordination in your movements?) to provide convergent validity of our speech measures as measuring motor difficulties.

#### Convergent validity: Finger-tapping scores

Participants also completed a computerized finger-tapping task, a well-established neuropsychological measure of motor deficits^7,59,92–98^. This is an ideal task because it taps into broad motor network function, including motor timing, which is often affected in motor speech disorders, has been found to be sensitive to mechanisms driving psychosis, and is readily amenable to reliable and valid in-person and remote assessments^7,26,92,99–110^. In this task, participants are instructed to press the spacebar with their index finger as quickly as possible for 10 s. They complete three trials per hand. Motivated by previous work^59,111^ and to parallel our speech measures, we study the coefficient of variation in number of taps across trials, calculated separately for the dominant and non-dominant hands.

#### Predictive validity: SIPS risk calculator

Finally, we use the SIPS-RC risk calculator^112^ to calculate a probability estimate of each participant’s risk of conversion to psychosis within one year (from SIPS and General Functioning scores^109^). SIPS-RC scores can range from 0.4% to 46.9%, but range from 0.8% to 10.1% in our sample.

### Adaptations to remote testing

Data collection occurred between 2019–2022, and our study had to be adapted to the remote format partway through due to the COVID-19 pandemic.

We adapted speech data collection, by mailing participants the same Zoom H2n recorders that had been used in the lab prior to the pandemic and having an experimenter administer the tasks over Zoom (tele-conferencing software). Similarly, all clinical interviews were conducted over Zoom beginning March 2020. Finally, the finger-tapping task, which, prior to March 2020, was collected in-lab as part of the Penn computerized neurocognitive battery^113^ and included 5 trials per hand was adapted into a shortened, online version, where participants only completed 3 trials per hand. To equate these measures, only the first 3 trials from each in-person participant’s task were used.

### Analyses

We run separate analyses for each speech measure in each speech sample type^114–119^. First, to test our prediction that CHR individuals exhibit more variability in their speech productions relative to controls, we run a linear regression predicting each speech measures (separately) from group status (CHR vs. HC). For durational speech measures, we control for averaged speech rate, by including it as an additional predictor in the regression. Next, for each speech measure that significantly differentiates clinical status, we test its clinical/convergent/predictive validity, by running separate linear regressions predicting each validation measure from each speech measure, within the CHR group only. We use an alpha level of $\alpha$ = 0.05 for all statistical tests.

### Supplementary information

Supplementary Materials for "Speech characteristics yield important clues about motor function. Speech variability in individuals at clinical high-risk for psychosis"

## Data Availability

All speech measure data and analysis code used in this study are available at github.com/khitczenko/chr_speech. The National Institute of Mental Health Data Archive provides the de-identified clinical, risk, and demographic information.
