# Supplementary material for: Speech characteristics yield important clues about motor function: Speech variability in individuals at clinical high-risk for psychosis
Source: Schizophrenia (Heidelb). 2023 Sep 16;9(1):60. doi: 10.1038/s41537-023-00382-9 (PMC10505148; doi:10.1038/s41537-023-00382-9)
Supplement: Supplementary file 1 — Supplementary Materials for "Speech characteristics yield important clues about motor function. Speech variability in individuals at clinical high-risk for psychosis" [file 41537_2023_382_MOESM1_ESM.pdf]

# Supplementary Materials for "Speech characteristics yield important clues about motor function. Speech variability in individuals at clinical high-risk for psychosis"

## Contents


**1 Methods** **1**
- 1.1 Full text of the Rainbow Passage . . . . . . 1
- 1.2 More information about automated speech analysis pipeline . . . . . . 2
- 1.3 Additional speech measures . . . . . . 3
- 1.4 Inspection of automated tool performance on our data . . . . . . 4

**2 Results** **7**
- 2.1 Additional results from the diadochokinetic speech tasks . . . . . . 7
- 2.2 Additional results from the read speech task . . . . . . 13
- 2.3 Additional results from the spontaneous speech task . . . . . . 15
- 2.4 Results studying variance of speech measures (instead of coefficients of variation) . . . . . . 16
- 2.5 Results studying mean speech measures . . . . . . 19
- 2.6 Categorization accuracy results . . . . . . 22
- 2.7 Results comparing the in-person and remote subgroups . . . . . . 25
- 2.8 Analyses studying the relationship between speech measures and demographics . . . . . . 27
- 2.9 Analyses studying the relationship between non-speech motor measures vs. symptom severity and risk of conversion . . . . . . 32
- 2.10 Analyses with a subset of participants removed . . . . . . 33

**3 Package and environment version** **38**


# 1 Methods

## 1.1 Full text of the Rainbow Passage

When the sunlight strikes raindrops in the air, they act as a prism and form a rainbow. The rainbow is a division of white light into many beautiful colors. These take the shape of a long round arch, with its path high above, and its two ends apparently beyond the horizon. There is, according to legend, a boiling pot of gold at one end. People look, but no one ever finds it. When a man looks for something beyond his reach, his friends say he is looking for the pot of gold at the end of the rainbow. Throughout the centuries people have explained the rainbow in various ways. Some have accepted it as a miracle without physical explanation. To the Hebrews it was a token that there would be no more universal floods. The Greeks used to imagine that it was a sign from the gods to foretell war or heavy rain. The Norsemen considered the rainbow as a bridge over which the gods passed from earth to their home in the sky. Others have tried to explain the phenomenon physically. Aristotle thought that the rainbow was caused by reflection of the sun's rays by the rain. Since then physicists have found that it is not reflection, but refraction by the raindrops which causes the rainbows. Many complicated ideas about the rainbow have been formed. The difference in the rainbow depends considerably upon the size of the drops, and the width of the colored band increases as the size of the drops increases. The actual primary rainbow observed is said to be the effect of superimposition of a number of bows. If the red of the second bow falls upon the green of the first, the result is to give a bow with

an abnormally wide yellow band, since red and green light when mixed form yellow. This is a very common type of bow, one showing mainly red and yellow, with little or no green or blue.

## 1.2 More information about automated speech analysis pipeline

### 1.2.1 Diadochokinetic Speech Samples

From each participant's two diadochokinetic speech samples, we automatically extracted: (1) the voice-onset-time for every syllable-initial voiceless stop consonant, (2) the duration of every vowel, (3) the first and second formants for every vowel (at 20% and midpoint of the vowel), as well as (4) the speech rate for each individual Diadochokinetic trial.

To do so, we used DDKtor (Segal et al., 2022; https://github.com/MLSpeech/DDKtor) to automatically obtain the VOTs and vowel durations, given hand-selected windows of analysis which corresponded to the individual Diadochokinetic trials. We then used FastTrack (Barreda, 2021; https://github.com/santiagobarreda/FastTrack) to obtain formants for every vowel that was identified by DDKtor. Finally, we obtained speech rate measures for each DDK trial directly from the DDKtor output. This was calculated as the number of syllables the participant produced in the trial divided by the amount of time it took them to produce it (i.e., the time elapsed from the start of the first syllable to the end of the last syllable, as found by DDKtor). We followed all recommendations and default settings given by the creators of the tools. In particular, following DDKtor recommendations, we excluded the shortest 2% and longest 5% of VOTs and vowel durations from our analyses and we double counted extremely long syllables (i.e., syllables with vowels that were at least twice as long as that speaker's average vowel duration) in speech rate calculations. FastTrack requires specifying a plausible range for the maximum analysis frequency value, which it uses to identify the best value below which it should search for F1-F5. Following FastTrack default recommendations, we set this range to 4500-6500 Hz for male speakers and 5000-7000 Hz for female speakers.

Although participants were asked to produce a specific number of syllables and trials, they sometimes deviated from this number. To ensure our measures were based on a sufficient, and relatively equal number of syllables/trials across participants, we excluded AMR (papapa/tatata/kakaka) trials in which participants produced fewer than 10 syllables (when they were supposed to produce 15) and SMR (pataka/katapa) trials in which participants produced fewer than 15 syllables (when they were supposed to produce 30). In addition, we excluded any extra trials participants produced (i.e., we capped the number of analyzed trials at 2 for each type of AMR trial - papapa, tatata, kakaka - and 10 for each type of SMR trial - pataka, katapa).

### 1.2.2 Read Speech Samples

From each participant's read speech sample, we extracted: (1) the voice-onset-time of every word-initial stop that preceded a vowel (voiced and voiceless), (2) the duration of every vowel with primary stress, (3) the first and second formants for all vowels with primary stress (at 20% and midpoint of the vowel), and (4) local speech rates.

To do so, we first obtained a transcript for each audio file, by manually editing the Rainbow Passage transcript to incorporate any participant speech errors or disfluencies. All meta comments on the task (e.g., "I think that's how you pronounce that' ') were removed. We then force-aligned the transcript to the audio files using the pre-trained Montreal Forced Aligner (McAuliffe et al., 2017; https://github.com/MontrealCorpusTools/Montreal-Forced-Aligner).

Given the output phone alignments, we obtained the VOTs of every word-initial stop consonant that preceded a vowel using AutoVOT, an automatic software for VOT detection (Adi et al., 2016; https://github.com/mlml/autovot). To best fit our data, we retrained a separate voiceless and voiced classifier using ~100 manually-annotated voiceless VOTs and ~100 manually-annotated voiced VOTs directly from our data. AutoVOT requires a window of analysis for every stop: per AutoVOT recommendations, we used the stop consonant boundaries output by the Montreal Forced Aligner, but extended by 31ms in both directions for voiceless stops and extended by 11ms in both directions for voiced stops. We obtained vowel durations directly from the Montreal Forced Aligner output phone alignments, by extracting the duration of all relevant vowels. Finally, we obtained formants (F1 and F2 at 20% and 50%) of each relevant vowel, by applying

FastTrack to all vowels bearing primary stress, as identified by the Montreal Forced Aligner. As with the DDK data, we set the frequency analysis range to 4500-6500 Hz for male speakers and 5000-7000 for female speakers.

Finally, we obtained a measure of the local speech rate of each phrase the participants produced. We followed Stuart-Smith et al. (2015) in defining local speech rate as the number of syllables per second in a phrase, where a phrase is any interval between silences that were at least 150ms long.

#### 1.2.3 Spontaneous Procedural Description: Peanut Butter and Jelly

Just as for the read speech sample, we extracted: (1) the voice-onset-time of every word-initial stop that preceded a vowel, (2) the duration of every vowel with primary stress, (3) the first and second formants for all vowels with primary stress (at 20% and 50% of the vowel), and (4) local speech rates. The process for extracting these values was identical to that for the Rainbow Passage, with the exception that the speech transcript was created from scratch, rather than by editing an existing transcript that participants read.

### 1.3 Additional speech measures

#### 1.3.1 Variability and overlap in vowel formants

In addition to the vowel duration measure discussed in the main text, we also analyzed variability in the first two vowel formants (the lowest two frequencies that have significant concentrations of energy around them; at a high-level, these measure what vowel was produced - e.g., /i/, /u/, etc.; Hillenbrand, 1995; Peterson & Barney, 1952).

- **Formant dispersion at 20% of the vowel (all speech samples)**: The first measure of vowel formant variability was calculated in two-dimensional (Formant1, Formant2) space as follows:

  $$\text{Formant Dispersion} = \frac{\sum_{i=1}^{n} \sqrt{(F1_i - F1_m)^2 + (F2_i - F2_m)^2}}{n}$$

  That is, we calculate the distance between each vowel token, $i$ ($i = 1...n$) with formants $F1_i$,$F2_i$), and the center of the vowel space (calculated as the thodsdian $F1$,$F2$: $F1_m$,$F2_m$). We then average these $n$ values to arrive at an average formant dispersion (Niziolek & Kiran, 2018). We chose to measure this early in the vowel (at 20% of its duration), as past work has shown that motor control issues are more likely to affect early production (i.e., before there is time to reach the target pronunciation). If anything, we would expect this choice to over-exaggerate differences in production related to motor control issues.

- **Change in formant dispersion between 20% and 50% of the vowel (all speech samples)**: The second measure of vowel formant variability was calculated as the difference between the average formant dispersions at 20% and 50% of the vowels:

  $$\Delta\text{Formant Dispersion} = \text{Formant Dispersion at 20\%} - \text{Formant Dispersion at 50\%}$$

  This measure provides a glimpse into how much the variability in formants changes over the course of the vowel, or how much the pronunciation needs to be adjusted to reach the target pronunciation by midpoint of the vowel (Niziolek & Kiran, 2018). Unlike other measures, this was not log-transformed in our analyses, as it can be negative or zero.

- **Overlap in vowel categories (read and spontaneous speech samples only)**: With increased variability in how vowels are produced, we would expect to see increased overlap between different vowel categories. To test this, we studied how much overlap there was between vowel tokens from different categories, using a measure of phonetic competition (sometimes also referred to as "repulsive force"; McCloy et al., 2015; Wright, 2004; Xie & Myers, 2018). More specifically, this is calculated as the sum of inverse squared distances between all pairs of vowel tokens coming from different vowel categories:

  $$\text{Phonetic Competition} = \sum_{i=1}^{n-1} \sum_{j=n+1}^{n} \frac{1}{(F2_i - F2_j)^2 + (F2_i - F2_j)^2}, \text{where } /i/ \neq /j/$$

where $i$ and $j$ represent two vowel tokens being compared, $/i/$ and $/j/$ represent their respective vowel categories, and $F1_i$ and $F2_i$ represent the first and second formants of vowel token $i$, respectively. At a high-level, phonetic competition measures how much different vowel categories overlap in their productions, with higher phonetic competition values corresponding to greater overlap. Because participants only produced one vowel type in the diadochokinetic speech task, this measure could only be calculated for the read and spontaneous speech samples.

#### 1.3.2 Pausing and Timing Measures

- **Coefficient of variation of syllable duration (diadochokinetic speech samples only)**: In addition to coefficients of variation at the consonant and vowel level individually, we also calculated the coefficient of variation over Consonant-Vowel syllable durations (e.g., "pa", "ta", "ka") in the Diadochokinetic speech task.
- **Coefficient of variation of intersyllable duration (diadochokinetic speech samples only)**: Intersyllable duration is the amount of time between the end of one syllable and the start of the next. This provides a measure of how much variability there is in the duration of pauses in the Diadochokinetic Speech task (Lozano-Goupil et al., 2022). If there is low variability, then the participants are producing the syllables at a nearly constant rate. If there is high variability, then they are not.
- **Total number of pauses (normalized by number of words produced; read and spontaneous speech samples only)**: We calculated the number of pauses, defined as any silence longer than 150ms. We divided this number by the total number of words the participant produced, to account for differences in total speech sample duration (this could differ even in the Read speech, as participants sometimes stumbled over words, repeated fragments, overlooked words, etc.). Note we excluded this measure for the Diadochokinetic Speech task, as the number of pauses was pre-specified by the nature of the task. Pausing has previously been studied in the high-risk group by Sichlinger et al. (2019) and Stanislawski et al. (2021).

Note: We also studied a number of other speech measures that we do not discuss or report detailed results for here. In particular, we also studied pairwise overlap measures between individual stop categories (e.g., /p/-/t/), finding null results, mirroring the vowel overlap measures presented here. With regards to validation measures (i.e., non-speech motor measures), we also studied the SMAP-R scale (Sensorimotor and Activity Psychosis-Risk score; Damme et al., 2021), but only had this measure for ~30 participants and found null results, mirroring the results reported here. Finally, in post-hoc exploratory analyses, we also studied mean and variance speech measures, which we report on in SM 2.4 and SM 2.5.

### 1.4 Inspection of automated tool performance on our data

The creators of the automated tools we use report high levels of reliability. However, to further validate these tools, we investigated the predicted speech measure values on our collected speech samples and compared them against established norms. Overall, we found that the predicted speech measure values matched expected average values, thus providing further evidence of the reliability of the automated tools we use.

#### 1.4.1 DDKtor: Voice-Onset-Times

As reported in the main text, the DDKtor software matches human annotations of diadochokinetic segment duration with correlations of $r$=0.85-0.90 and matches human annotation of diadochokinetic speech rate with correlations of $r$=0.94-0.97. The plotted density distributions for voiceless voice-onset-times (output from DDKtor on our collected diadochokinetic speech samples; Figure S1) match expectations, and provide additional evidence of DDKtor's reliability.

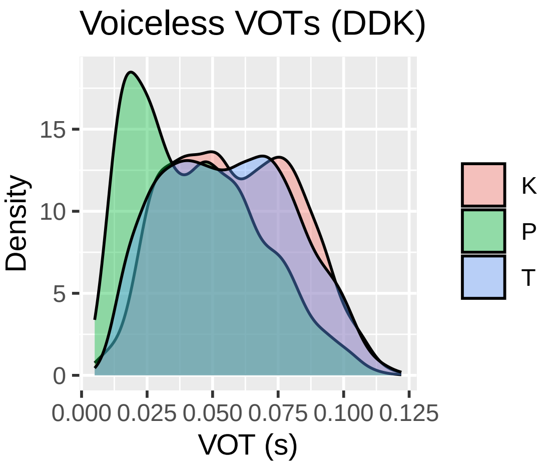


Figure S1: Density distribution of voiceless stop voice-onset-times (VOTs) in the diadochokinetic (DDK) speech samples, as output from the DDKtor software.

### 1.4.2 AutoVOT: Voice-Onset-Times

As reported in the main text, the AutoVOT software, which predicts voice-onset-times, parallels interrater reliability rates, with ~90% of its predicted voice-onset-times are within 10-15ms of gold-standard human annotation. Figure S2 shows the predict voice-onset-times for vowels in our collected read (top) and spontaneous (bottom) speech samples. As with DDKtor, this plot provides further evidence of AutoVOT's reliability, as the density plots match expectations based on previously-established norm voice-onset-time values.

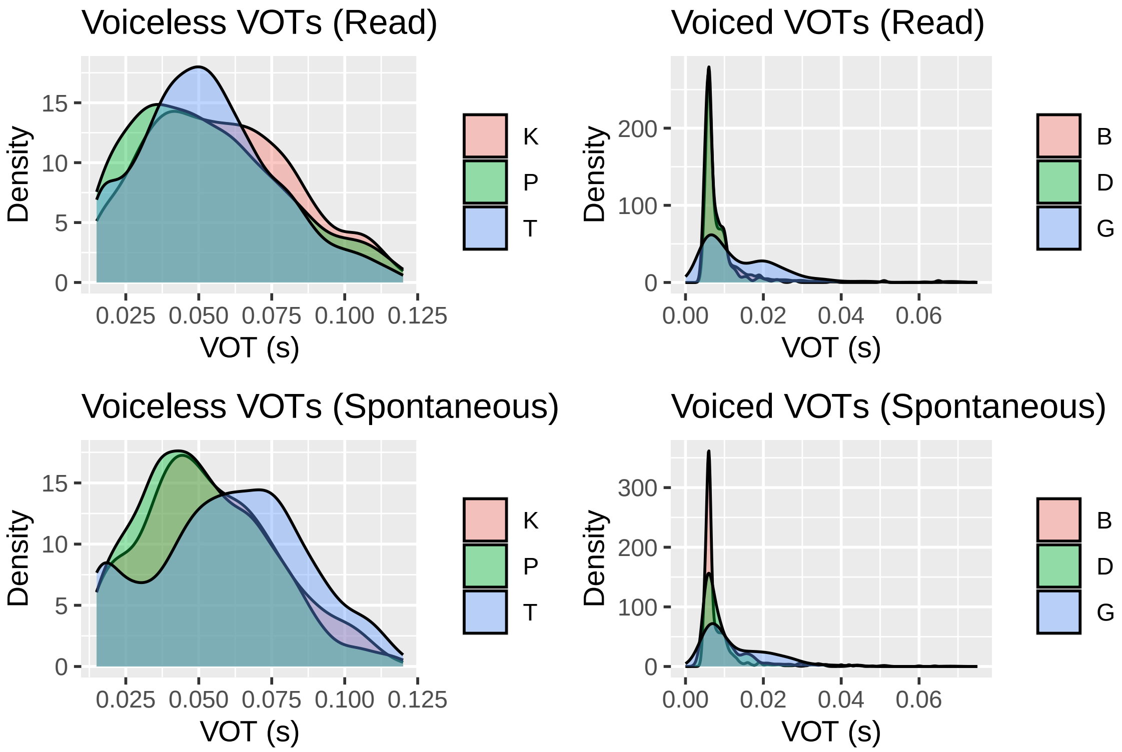


Figure S2: Density distribution of voiceless and voiced stop voice-onset-times (VOTs) in the read (top) and spontaneous (bottom) speech samples, as output from the AutoVOT software.

Table S 1: Comparison of average predicted F1/F2 values for the read and spontaneous speech subtasks (combined for male/female participants) to reference average values from Hillenbrand et al. (1995), split by sex. Spont. = Spontaneous; Ref. = Reference; Fem. = Female

| Vowel | Read F1 | Read F2 | Spont. F1 | Spont. F2 | Ref Male F1 | Ref Male F2 | Ref Fem F1 | Ref Fem F2 |
|---|---|---|---|---|---|---|---|---|
| AA1 | 737 | 1285 | 698 | 1374 | 768 | 1333 | 936 | 1551 |
| AE1 | 684 | 1748 | 683 | 1880 | 588 | 1952 | 669 | 2349 |
| AH1 | 617 | 1379 | 657 | 1403 | 623 | 1200 | 753 | 1426 |
| AO1 | 579 | 1120 | 608 | 1144 | 652 | 997 | 781 | 1136 |
| EH1 | 608 | 1764 | 624 | 1765 | 580 | 1788 | 731 | 2058 |
| ER1 | 512 | 1511 | 523 | 1528 | 474 | 1379 | 523 | 1588 |
| EY1 | 522 | 2079 | 488 | 2187 | 476 | 2089 | 536 | 2530 |
| IH1 | 473 | 1873 | 485 | 1923 | 427 | 2034 | 483 | 2365 |
| IY1 | 389 | 2277 | 400 | 2334 | 342 | 2322 | 437 | 2761 |
| OW1 | 547 | 1199 | 523 | 1297 | 497 | 910 | 555 | 1035 |
| UH1 | 512 | 1323 | 476 | 1683 | 469 | 1122 | 519 | 1225 |
| UW1 | 405 | 1678 | 390 | 1842 | 378 | 997 | 459 | 1105 |

#### 1.4.3 FastTrack: Vowel Formants

FastTrack is reported to have an average error of ~20Hz and 98.9% of vowels have errors of less than 5% off from the human-annotated value. We inspected the performance of FastTrack on our data, by visualizing the extracted values and comparing the average value extracted from our data to the average formant values reported for American speakers by Hillenbrand et al. (1995). Figure S3 shows the predicted F1/F2 formant values by speech task, and Table S1 shows the average F1/F2 formant values by speech task (combined for male/female participants) side-by-side with those reported in Hillenbrand et al. (1995), separated by speaker sex. While we do not include diadochokinetic speech in Table S1 (as participants only produced one vowel type in this task), the average formant value output by FastTrack on that task (F1: 687; F2: 1452) also closely matched average values reported by Hillenbrand et al. (1995). Overall, these results provide confidence that the extracted formant values are reliable, across all speech sample types that we study.

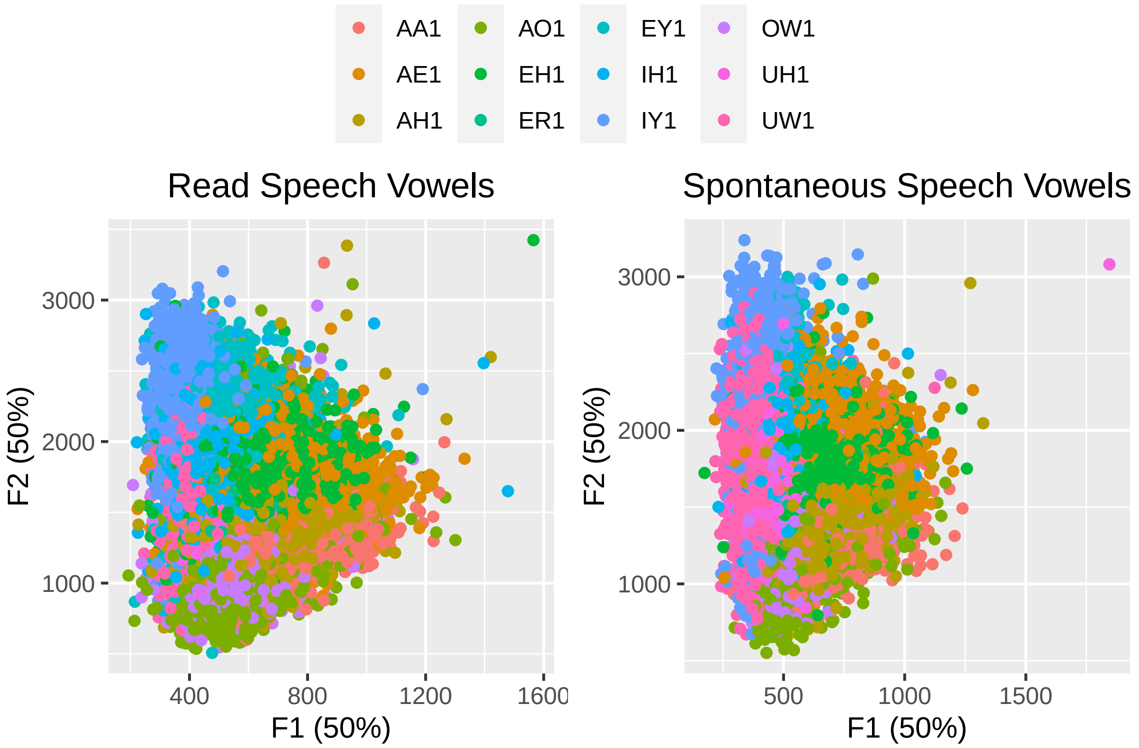


Figure S3: F1/F2 values predicted by FastTrack for each vowel in our Read speech (left) and Spontaneous speech (right) samples, colored by their vowel type. Overall, the F1/F2 values appear valid and reliable across both speech sample types.

# 2 Results

## 2.1 Additional results from the diadochokinetic speech tasks

### 2.1.1 Clinical High-Risk vs. Healthy Control group differences

Here, we provide details of analyses of all acoustic speech features tested that were not discussed in the main paper (Figure S4). In particular, none of the following other speech measures significantly **differed between** the CHR vs. HC groups in either the SMR ("pataka") or AMR ("papapa") subtasks: coefficient of variation of vowel duration (AMR: $\beta$ = 0.12, s.e. = 0.08, t = 1.52, p = 0.132; SMR: $\beta$ = 0.1, s.e. = 0.07, t = 1.35, p = 0.179), change in formant dispersion between 20% and 50% of the vowel (AMR: $\beta$ = 3.9, s.e. = 4.02, t = 0.97, p = 0.334; SMR: $\beta$ = 0.92, s.e. = 3.85, t = 0.24, p = 0.811), coefficient of variation of syllable duration (AMR: $\beta$ = 0.14, s.e. = 0.09, t = 1.63, p = 0.107; SMR: $\beta$ = 0.1, s.e. = 0.06, t = 1.66, p = 0.101), and coefficient of variation of intersyllable duration (AMR: $\beta$ = 0.2, s.e. = 0.1, t = 2, p = 0.048; SMR: $\beta$ = 0.09, s.e. = 0.06, t = 1.37, p = 0.174), though some approached significance. Average formant dispersion at 20% of the vowel significantly predicted CHR vs HC group in the AMR subtask ($\beta$ = 0.21, s.e. = 0.1, t = 2.17, p = 0.033), but not the SMR (though it approached significance: $\beta$ = 0.14, s.e. = 0.08, t = 1.76, p = 0.081).

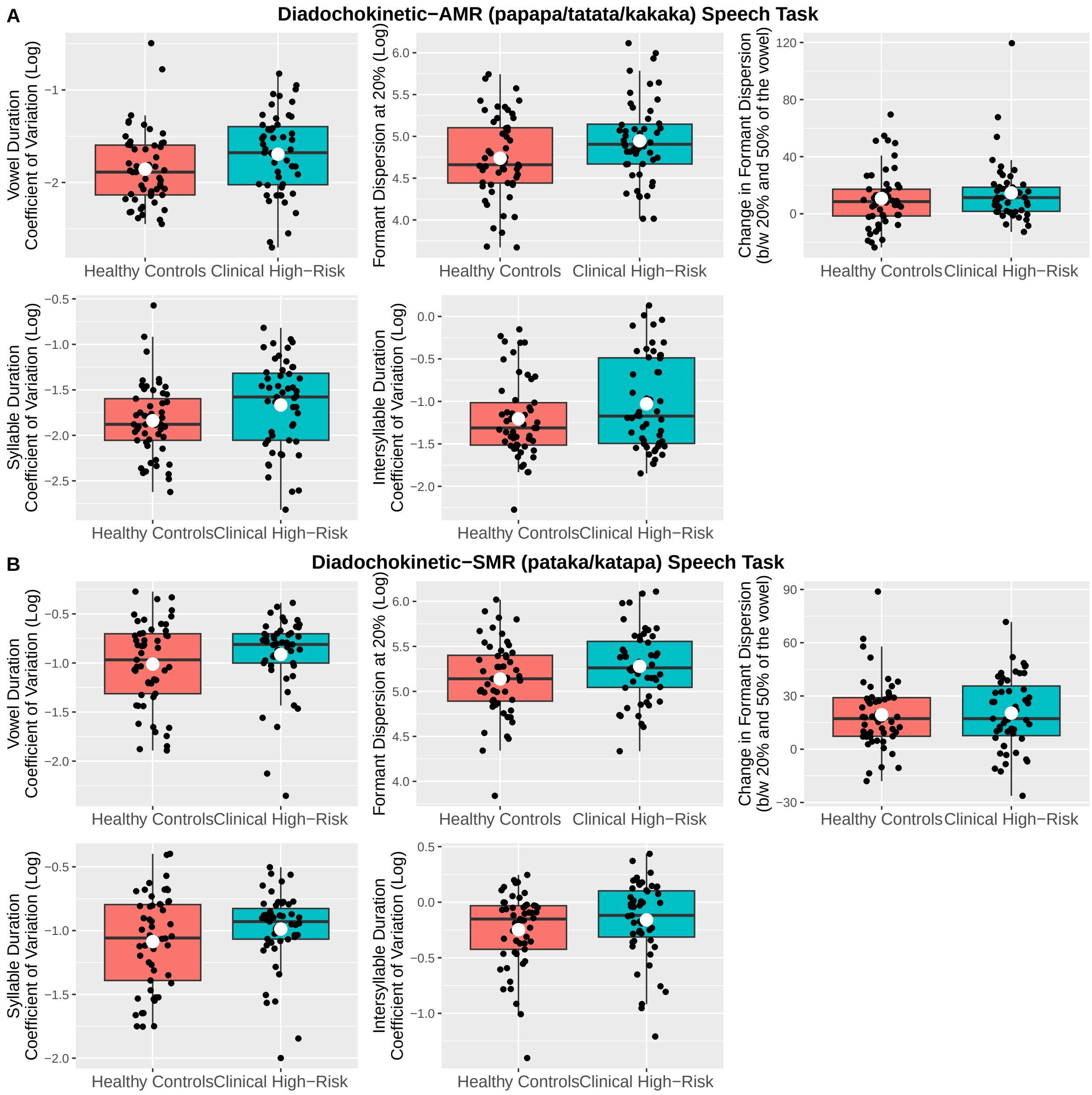


Figure S4: CHR vs HC group comparisons for vowel and pausing/timing speech measures in the (A) Diadochokinetic AMR and (B) Diadochokinetic SMR subtasks. In addition to the significant results reported in the main text, we found that average formant dispersion at 20% of the vowel was significantly higher in the CHR group than the HC group in the Diadochokinetic-AMR subtask, but we observed no other significant group differences. Each black dot represents one participant; the white dot represents the average value across participants.

### 2.1.2 Correlations with clinical/motor/risk variables

Next, we correlated the measures that consistently differentiated the CHR vs HC groups (i.e., variability in voiceless stop voice-onset-times and variability in speech rates) against SIPS scores, finger tapping scores, and risk scores, but generally found no significant correlations in either the AMR or SMR speech samples.

**2.1.2.1 Variability in voiceless voice-onset-times** Results are presented in Figure S5. We found that variation in the voiceless voice-onset-times did not correlate with SIPS total positive symptoms scores (AMR: $\beta$ = 0.1, s.e. = 1.99, t = 0.05, p = 0.962; SMR: $\beta$ = -1.08, s.e. = 1.99, t = -0.54, p = 0.591), SIPS total negative symptoms scores (AMR: $\beta$ = 4.89, s.e. = 3.08, t = 1.59, p = 0.119; SMR: $\beta$ = 4.66, s.e. = 3.08, t = 1.51, p = 0.137), SIPS total disorganized symptoms scores (AMR: $\beta$ = 0.63, s.e. = 1.43, t = 0.44, p = 0.659; SMR: $\beta$ = 2.6, s.e. = 1.52, t = 1.71, p = 0.094), or an individual SIPS item "motor difficulties" (G3; AMR: $\beta$ = -0.49, s.e. = 0.58, t = -0.85, p = 0.398; SMR: $\beta$ = 0.61, s.e. = 0.63, t = 0.97, p = 0.338). We also found that variation in the voiceless voice-onset-times did not relate to non-speech motor measures, namely, the coefficient of variation of finger tapping in the dominant (AMR: $\beta$ = 0.11, s.e. = 0.42, t = 0.27, p = 0.787; SMR: $\beta$ = 0.22, s.e. = 0.46, t = 0.47, p = 0.639) and non-dominant hands (AMR: $\beta$ = 0, s.e. = 0.38, t = 0.01, p = 0.991; SMR: $\beta$ = 0.13, s.e. = 0.43, t = 0.3, p = 0.769). Finally, we found that variation in the voiceless voice-onset-times did predict SIPS-RC risk of conversion scores in the AMR ("papapa"/"tatata"/"kakaka") subtask ($\beta$ = 2.75, s.e. = 1.18, t = 2.32, p = 0.025, but not the SMR ("pataka"/"katapa") subtask ($\beta$ = 1.91, s.e. = 1.35, t = 1.41, p = 0.165).

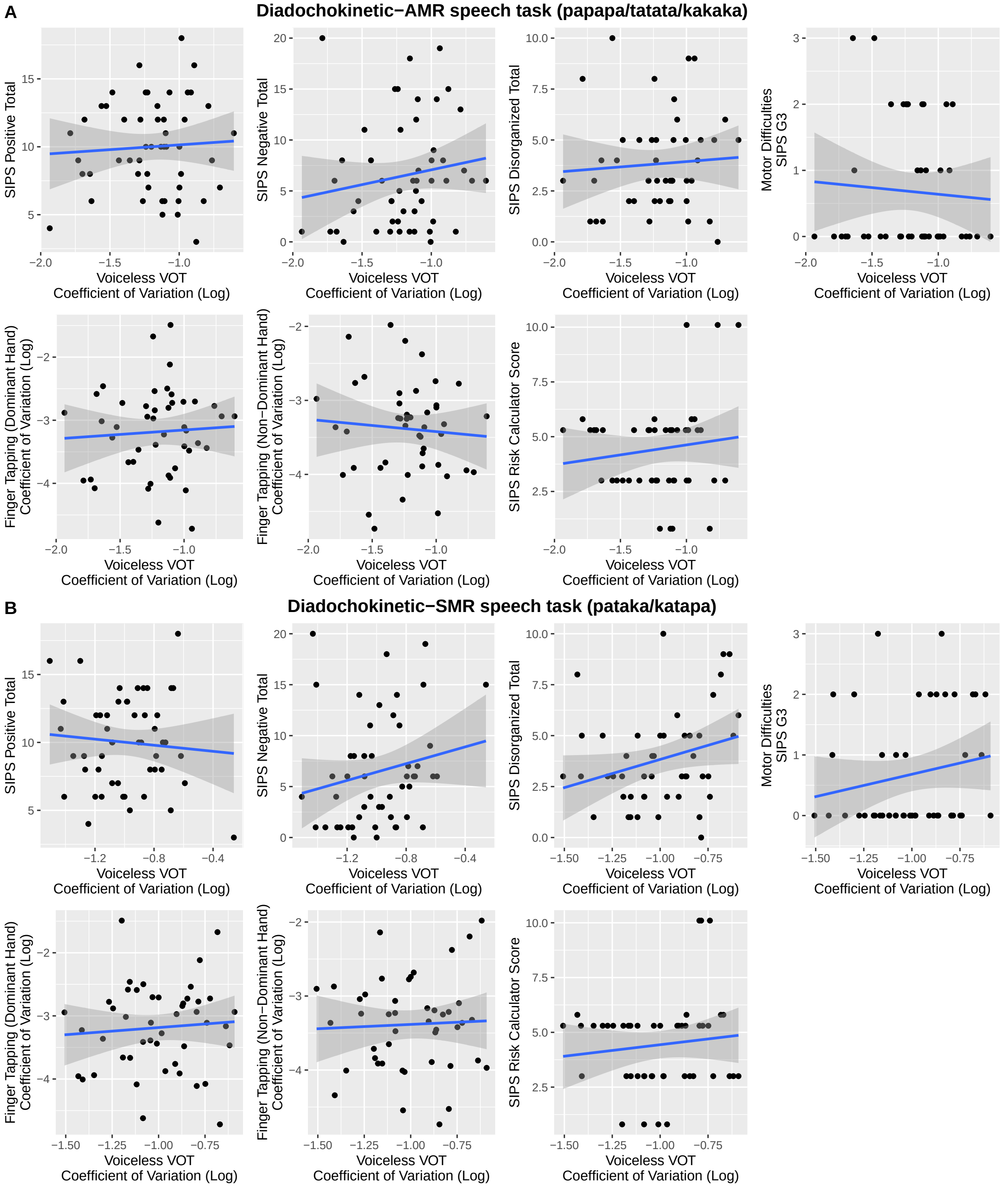


Figure S5: Variability in voiceless stop production vs. SIPS (symptom) scores, finger-tapping (motor) scores, and SIPS-RC risk of conversion scores in the (A) Diadochokinetic-AMR subtask and (B) Diadochokinetic-SMR subtask. Variability in voiceless stop production was positively correlated with risk scores in the AMR subtask, but showed no significant correlations with any other measures in either subtask. Each point represents one participant; the line represents the line of best-fit, with shaded regions showing standard errors of the regression fit.

**2.1.2.2 Variability in speech rate** Results are presented in Figure S6. Similarly, contrary to predictions, we found that the coefficient of variation in speech rate (which was shown to differ between the Clinical High-Risk and Healthy Control groups) also did not correlate with symptom severity (as measured by the SIPS), non-speech motor measures (as measured by Finger Tapping scores), or risk of conversion to psychosis scores (SIPS-RC). In particular, we found that variation in speech rate did not correlate with SIPS total positive symptoms scores (AMR: $\beta$ = 1.16, s.e. = 0.87, t = 1.32, p = 0.192; SMR: $\beta$ = -0.52, s.e. = 1.04, t = -0.5, p = 0.618), SIPS total negative symptoms scores (AMR: $\beta$ = -0.67, s.e. = 1.4, t = -0.48, p = 0.636; SMR: $\beta$ = -2.08, s.e. = 1.62, t = -1.28, p = 0.205), SIPS total disorganized symptoms scores (AMR: $\beta$ = 1.19, s.e. = 0.66, t = 1.81, p = 0.077; SMR: $\beta$ = 0.67, s.e. = 0.73, t = 0.91, p = 0.366), or an individual SIPS item measuring "motor difficulties" (G3; AMR: $\beta$ = -0.2, s.e. = 0.28, t = -0.72, p = 0.477; SMR: $\beta$ = -0.12, s.e. = 0.3, t = -0.41, p = 0.682). We also found that variation in speech rate did not relate to non-speech motor measures, namely, the coefficient of variation of finger tapping in the dominant (AMR: $\beta$ = 0.11, s.e. = 0.2, t = 0.53, p = 0.602; SMR: $\beta$ = -0.13, s.e. = 0.22, t = -0.61, p = 0.542) and non-dominant hands (AMR: $\beta$ = 0.35, s.e. = 0.17, t = 2.01, p = 0.051; SMR: $\beta$ = 0.14, s.e. = 0.21, t = 0.67, p = 0.508). Finally, we found that variation in speech rate did not predict SIPS-RC risk of conversion scores (AMR: $\beta$ = 0.28, s.e. = 0.61, t = 0.45, p = 0.654; SMR: $\beta$ = -0.42, s.e. = 0.66, t = -0.64, p = 0.527).

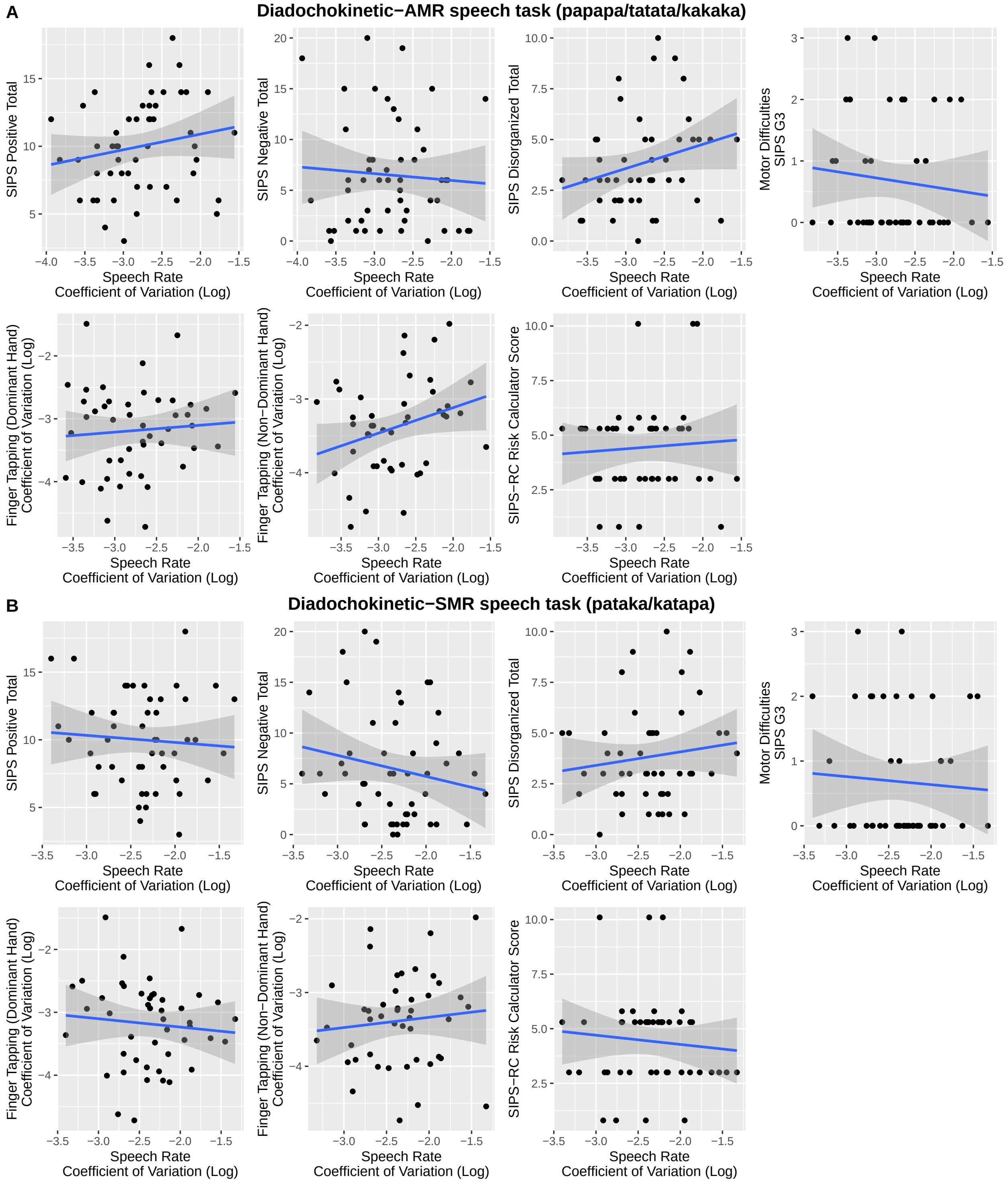


Figure S6: Variability in speech rate vs. SIPS (symptom) scores, finger-tapping (motor) scores, and SIPS-RC risk of conversion scores in the (A) Diadochokinetic-AMR subtask and (B) Diadochokinetic-SMR subtask. Variability in speech rate showed no significant correlations with any validation measures in either subtask. Each point represents one participant; the line represents the line of best-fit, with shaded regions showing standard errors of the regression fit.

## 2.2 Additional results from the read speech task

### 2.2.1 Clinical High-Risk vs. Healthy Control group differences

Results are presented in Figure S7. In addition to the analyses reported in the main text, we found that voiced consonant production, vowel production, and pausing did not differentiate Clinical High-Risk vs. Healthy Controls in our data. In particular, none of the following speech measures significantly predicted group status in the Rainbow Passage task: coefficient of variation of voiced stop consonants /b, d, g/ ($\beta$ = -0.03, s.e. = 0.07, t = -0.43, p = 0.667), coefficient of variation of vowel duration ($\beta$ = 0.01, s.e. = 0.02, t = 0.82, p = 0.417), average formant dispersion at 20% of the vowel ($\beta$ = 0.03, s.e. = 0.03, t = 0.99, p = 0.327), change in formant dispersion between 20% and 50% of the vowel ($\beta$ = -0.08, s.e. = 2.75, t = -0.03, p = 0.977), overlap between vowel categories, calculated as the phonetic competition between different vowel types ($\beta$ = 0.12, s.e. = 0.26, t = 0.46, p = 0.643), and number of pauses per word produced ($\beta$ = 0.08, s.e. = 0.05, t = 1.51, p = 0.133).

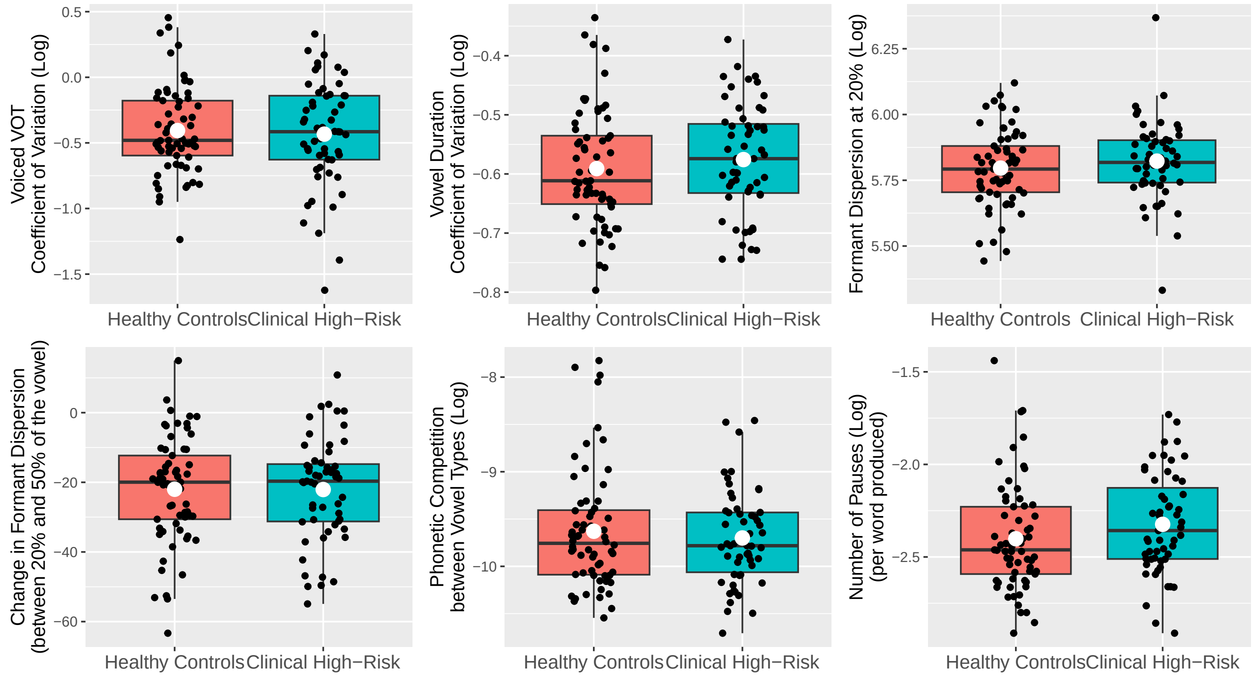


Figure S7: Other than those reported in the main text, we found no significant CHR vs HC group differences in speech measures in the reading task. This graph presents vowel and pausing/timing measures. Each black dot represents one participant; the white dot represents the average value across participants.

### 2.2.2 Correlations with clinical/motor/risk variables

Next, we tested whether the two speech measures that showed group differences (coefficient of variation in voiceless voice-onset-times and coefficient of variation in speech rate) correlated with clinical, non-speech motor, and risk variables. We predicted that increased variability in speech production would correlate with worse symptom severity, increased variability on other motor measures, and with a heightened risk of conversion to psychosis. However, just as in the Diadochokinetic speech task analyses, we found that these speech measures mostly did not correlate with motor, clinical, or risk measures.

**2.2.2.1 Variability in voiceless voice-onset-times** Results are presented in Figure S8. We found that variation in the voiceless voice-onset-times did not correlate with SIPS total positive symptoms scores ($\beta$ = -2.76, s.e. = 2.12, t = -1.3, p = 0.2), SIPS total negative symptoms scores ($\beta$ = -0.11, s.e. = 3.6, t = -0.03, p = 0.976), SIPS total disorganized symptoms scores ($\beta$ = 1.88, s.e. = 1.61, t = 1.17, p = 0.248), or an individual SIPS item measuring "motor difficulties" (G3; $\beta$ = -0.43, s.e. = 0.69, t = -0.62, p = 0.539).

We also found that variation in the voiceless voice-onset-times did not relate to non-speech motor measures, namely, the coefficient of variation of finger tapping in the dominant ($\beta$ = 0.41, s.e. = 0.46, t = 0.88, p = 0.386) and non-dominant hands ($\beta$ = -0.51, s.e. = 0.44, t = -1.14, p = 0.259). Finally, we found that variation in the voiceless voice-onset-times did not predict SIPS-RC risk of conversion scores ($\beta$ = 1.02, s.e. = 1.47, t = 0.7, p = 0.49).

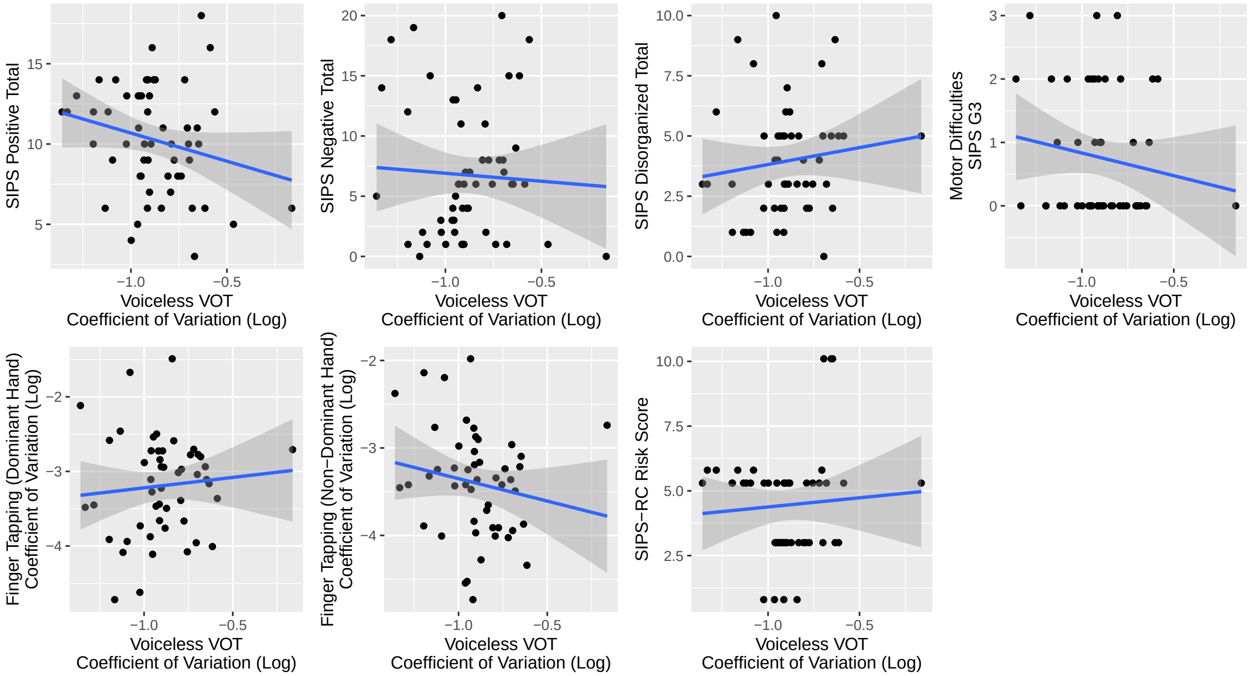


Figure S8: We observed no significant correlations between variability in voiceless voice-onset-time production vs. clinical/non-speech motor/risk variables in the Read speech task. Each point represents one participant; the line represents the line of best-fit, with shaded regions showing standard errors of the regression fit.

**2.2.2.2 Variability in speech rate** Results are presented in Figure S9. As reported in the main text, we found that variation in local speech rates did correlate with one of the non-speech motor measures, namely the coefficient of variation of finger tapping in the non-dominant hand ($\beta$ = 0.93, s.e. = 0.32, t = 2.86, p = 0.006), but not the dominant hand ($\beta$ = 0.28, s.e. = 0.37, t = 0.74, p = 0.465). However, as in the Diadochokinetic speech tasks, we found that variation in the local speech rates did not correlate with the other clinical and risk score measures we studied. In particular, we found that variation in the local speech rates did not correlate with SIPS total positive symptoms scores ($\beta$ = 0.89, s.e. = 1.78, t = 0.5, p = 0.62), SIPS total negative symptoms scores ($\beta$ = -1.13, s.e. = 2.95, t = -0.38, p = 0.702), SIPS total disorganized symptoms scores ($\beta$ = 0.18, s.e. = 1.29, t = 0.14, p = 0.89), or an individual SIPS item measuring "motor difficulties" (G3; $\beta$ = 0.43, s.e. = 0.56, t = 0.77, p = 0.448). We also found that variation in local speech rates did not predict SIPS-RC risk of conversion scores ($\beta$ = -0.77, s.e. = 1.16, t = -0.67, p = 0.507), mirroring the results from the Diadochokinetic speech tasks.

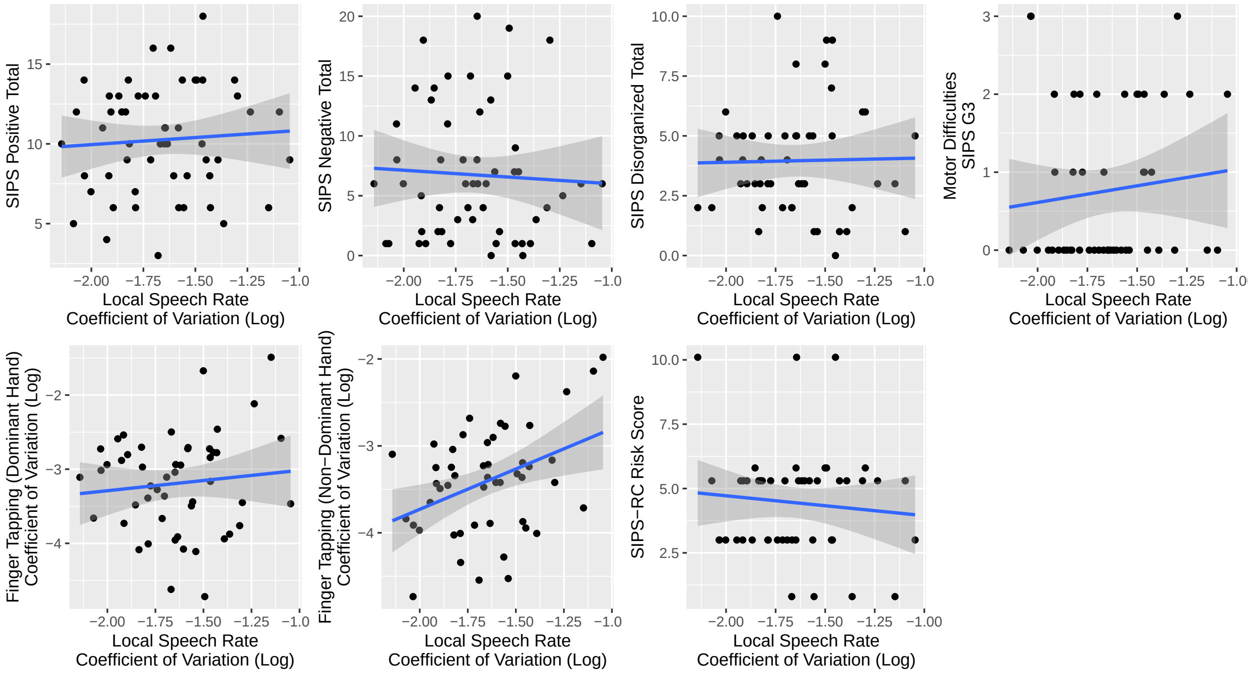


Figure S9: With the exception of finger tapping in the non-dominant hand, we observed no other significant correlations between variability in speech rate vs. clinical/non-speech motor/risk variables in the Read speech task. Each point represents one participant; the line represents the line of best-fit, with shaded regions showing standard errors of the regression fit.

## 2.3 Additional results from the spontaneous speech task

### 2.3.1 Clinical High-Risk vs. Healthy Control group differences

Results are presented in Figure S10. In addition to the null results reported in the main text, we found that voiced consonant production, vowel production, and pausing did not differentiate Clinical High-Risk vs. Healthy Controls in the procedural description task data. In particular, none of the following speech measures significantly predicted group status in the Peanut Butter & Jelly task: coefficient of variation of voiced stop consonants /b, d, g/ ($\beta$ = 0.15, s.e. = 0.11, t = 1.36, p = 0.177), coefficient of variation of vowel duration ($\beta$ = 0.01, s.e. = 0.03, t = 0.36, p = 0.718), average formant dispersion at 20% of the vowel ($\beta$ = -0.01, s.e. = 0.03, t = -0.3, p = 0.768), change in formant dispersion between 20% and 50% of the vowel ($\beta$ = -2.44, s.e. = 3.48, t = -0.7, p = 0.484), overlap between vowel categories, calculated as the phonetic competition between different vowel types ($\beta$ = 0.09, s.e. = 0.12, t = 0.76, p = 0.449), and number of pauses per word produced ($\beta$ = 0.07, s.e. = 0.08, t = 0.84, p = 0.403). Because none of the acoustic speech tasks showed significant results, we did not test for correlations with non-speech motor/clinical/risk measures.

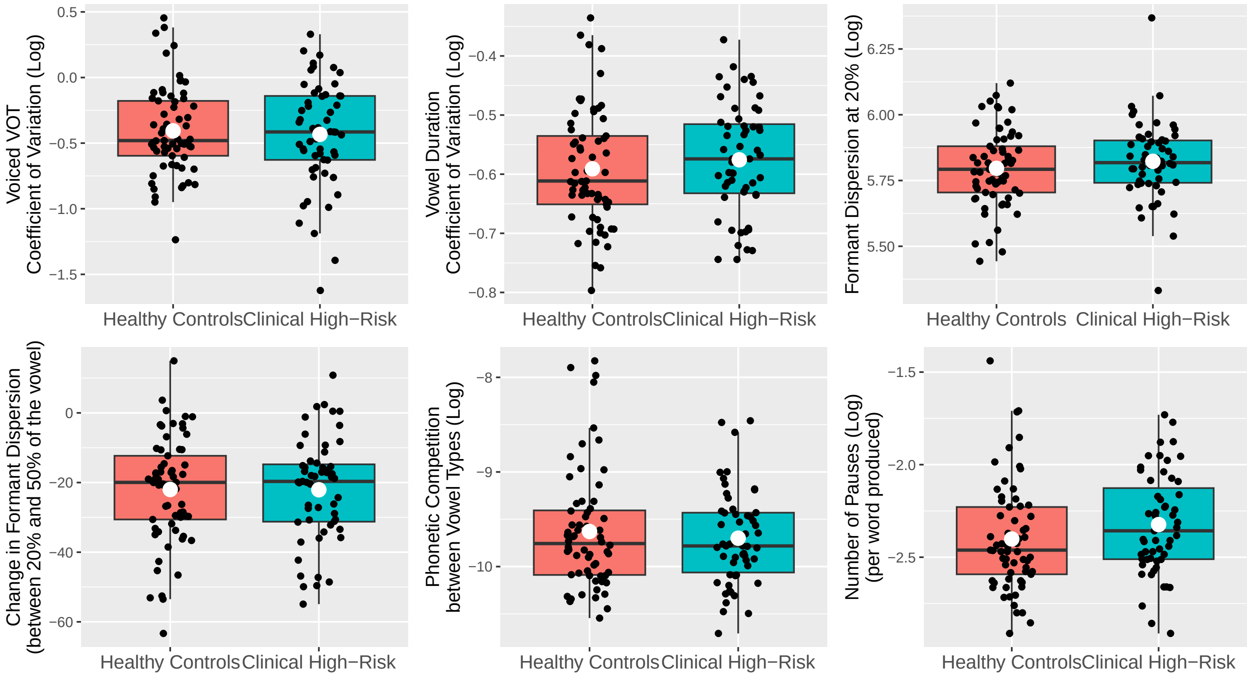


Figure S10: We found no significant CHR vs HC group differences in any speech measures in the spontaneous procedural description task. This graph presents vowel and pausing/timing measures. Each black dot represents one participant; the white dot represents the average value across participants.

## 2.4 Results studying variance of speech measures (instead of coefficients of variation)

### 2.4.1 Diadochokinetic Speech Tasks

Results are plotted in Figure S11. CHR participants exhibited significantly more variance in their voiceless stop consonant voice-onset-times than HC in the AMR trials ($\beta = 0.26$, s.e. = 0.12, t = 2.15, p = 0.034), and near-significantly more variance than HC in the SMR trials ($\beta = 0.22$, s.e. = 0.12, t = 1.91, p = 0.059). In addition, CHR participants produced more variance in their speech rates across both AMR ($\beta = 0.98$, s.e. = 0.31, t = 3.2, p = 0.002) and SMR trials ($\beta = 0.51$, s.e. = 0.25, t = 2.06, p = 0.041). All other following speech measures showed no group differences: variance in vowel duration (AMR: $\beta = 0.22$, s.e. = 0.15, t = 1.49, p = 0.139; SMR: $\beta = 0.17$, s.e. = 0.15, t = 1.1, p = 0.273), variance in syllable duration (AMR: $\beta = 0.28$, s.e. = 0.16, t = 1.73, p = 0.086; SMR: $\beta = 0.19$, s.e. = 0.13, t = 1.44, p = 0.153), and variance in intersyllable duration (AMR: $\beta = 0.41$, s.e. = 0.22, t = 1.86, p = 0.066; SMR: $\beta = 0.23$, s.e. = 0.17, t = 1.32, p = 0.191), mirroring what was observed with the corresponding coefficients of variation.

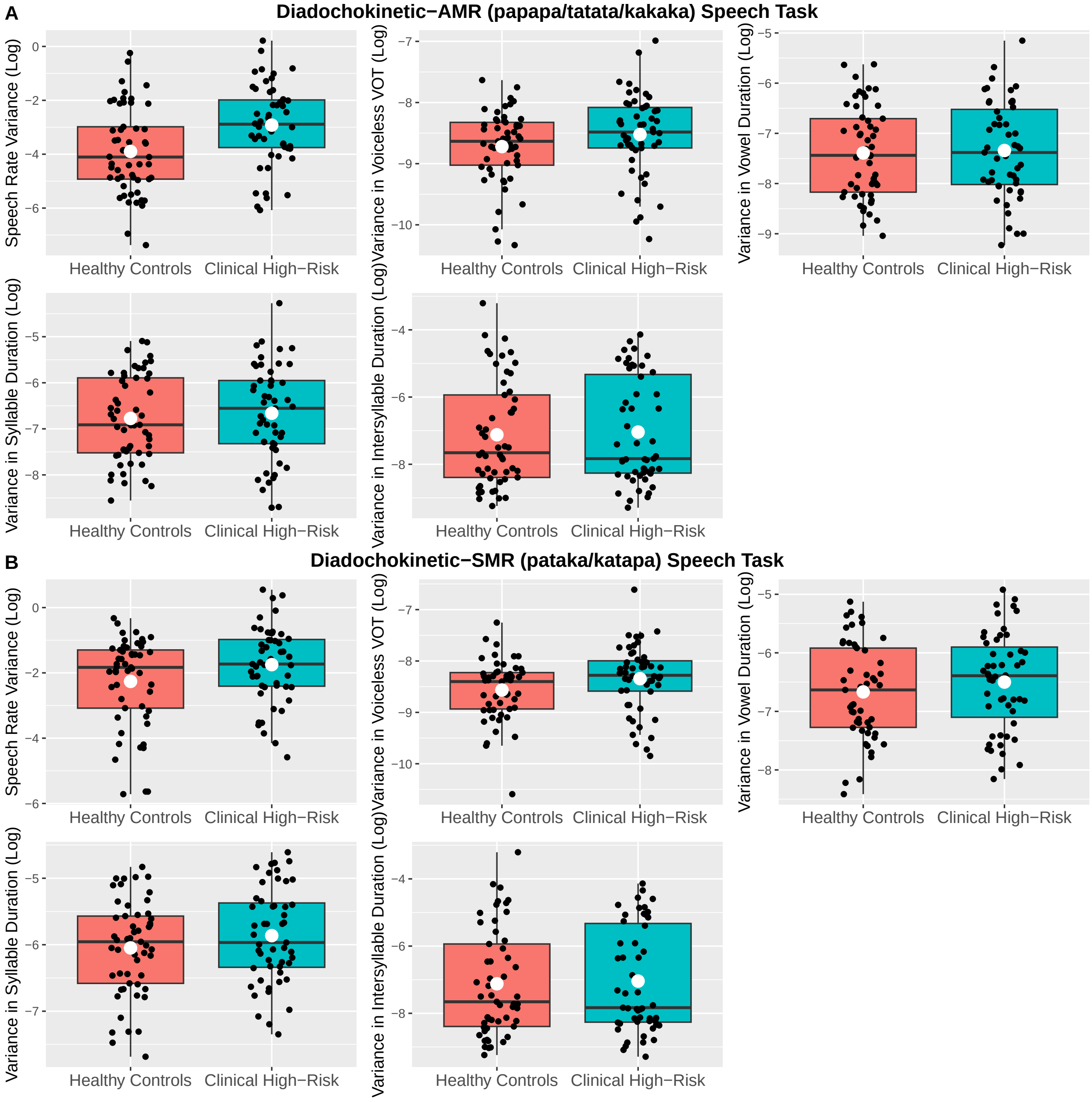


Figure S11: Group differences in speech measure *variance* in the (A) Diadochokinetic AMR or (B) Diadochokinetic SMR subtasks. Results largely mirror coefficient of variation results, with significant or near-significant group differences in speech rate variance and voiceless voice-onset-time variance. Each black dot represents one participant; the white dot represents the average value across participants.

### 2.4.2 Read Speech

In the read speech task, we found no significant CHR vs. HC group differences in variance in speech rate ($\beta$ = 0.16, s.e. = 0.09, t = 1.72, p = 0.087), variance in voiceless voice-onset-times ($\beta$ = 0.16, s.e. = 0.09, t = 1.68, p = 0.096), variance in voiced voice-onset-times ($\beta$ = -0.02, s.e. = 0.2, t = -0.12, p = 0.902), or variance in vowel durations ($\beta$ = 0.03, s.e. = 0.05, t = 0.64, p = 0.525) (Figure S12). However, we note that variance in speech rate and variance in voiceless voice-onset-times showed potential trends toward significance.

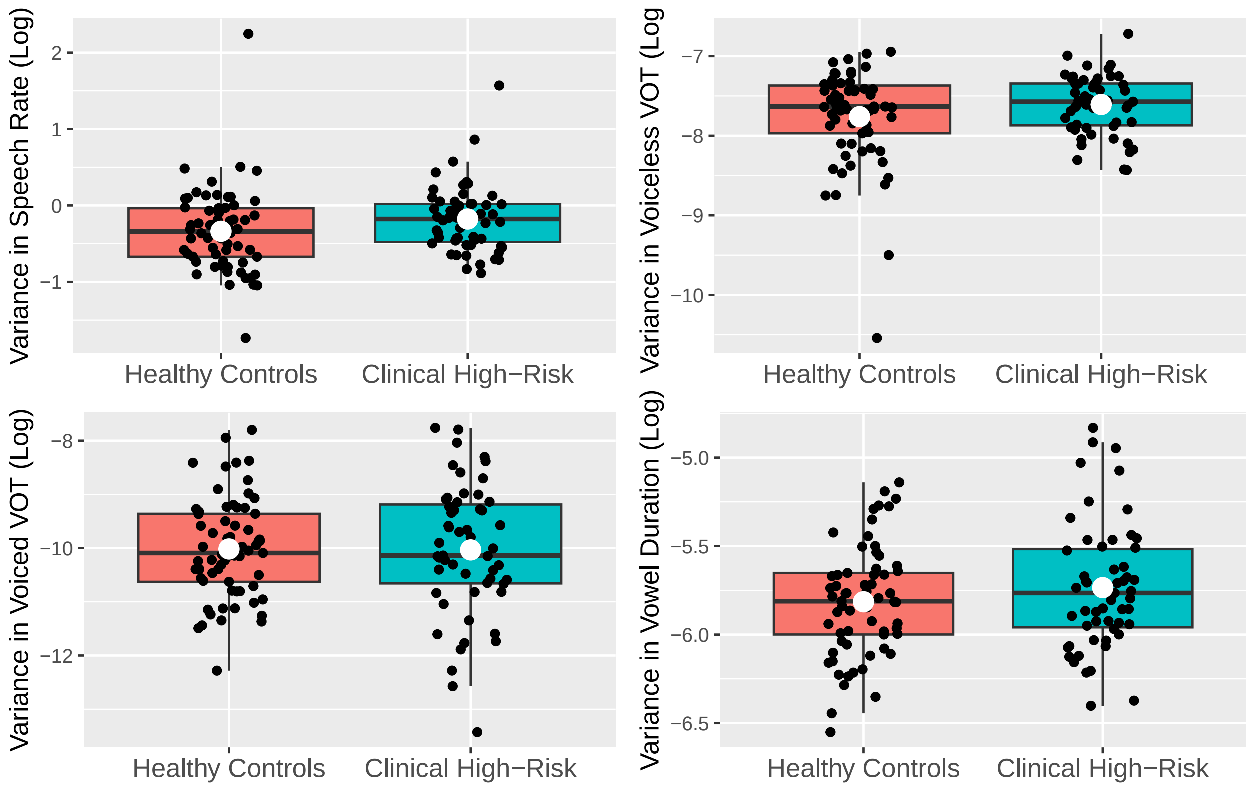


Figure S12: We find no significant CHR vs HC group differences in *variance* speech measures in the reading task, though differences in speech rate variance and voiceless voice-onset-time variance are trending towards significance. Each black dot represents one participant; the white dot represents the average value across participants.

### 2.4.3 Spontaneous Speech

Finally, in the spontaneous speech task, we found no significant CHR vs. HC group differences in speech rate variance ($\beta$ = 0.11, s.e. = 0.2, t = 0.55, p = 0.583), variance in voiceless voice-onset-times ($\beta$ = -0.07, s.e. = 0.1, t = -0.73, p = 0.468), variance in voiced voice-onset-times ($\beta$ = 0.37, s.e. = 0.31, t = 1.17, p = 0.245), or variance in vowel durations ($\beta$ = 0.01, s.e. = 0.08, t = 0.12, p = 0.905) (Figure S13), mirroring what we observed with coefficients of variation in the main text.

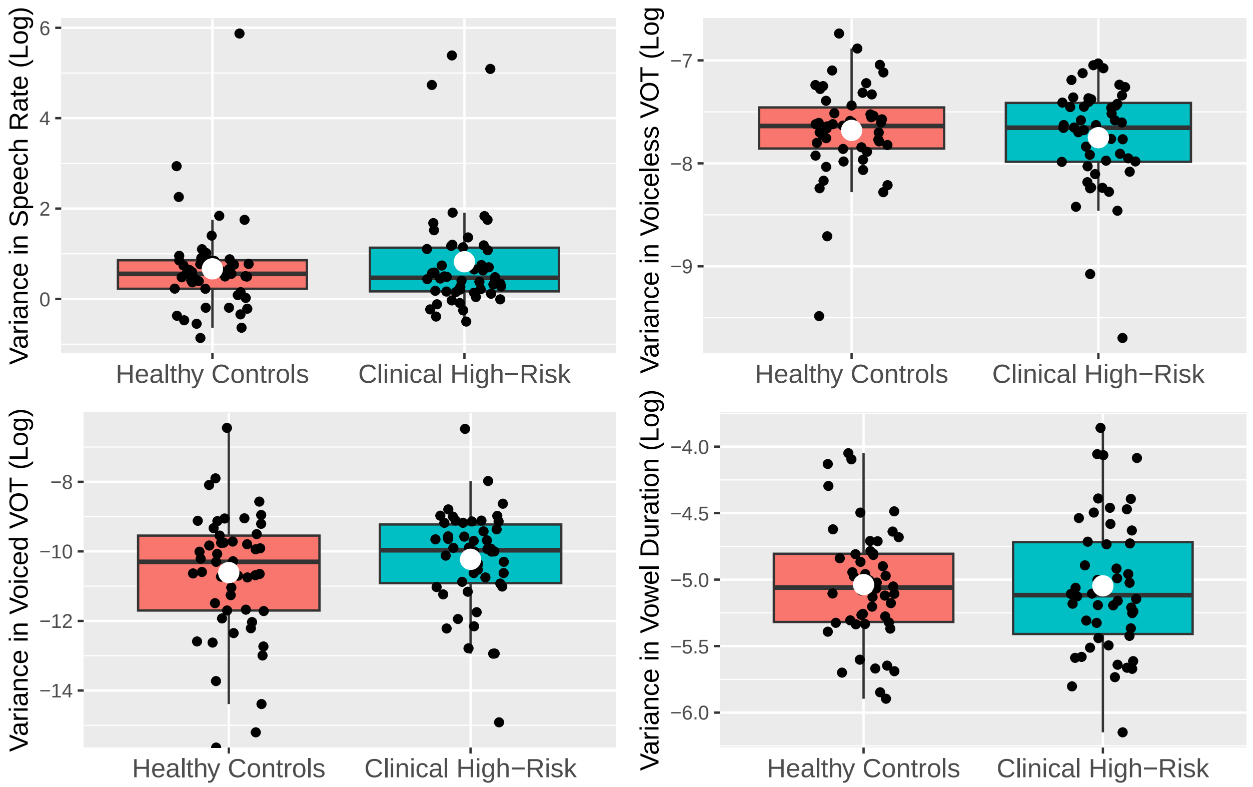


Figure S13: We find no significant CHR vs HC group differences in *variance* speech measures in the reading task, though differences in speech rate variance and voiceless voice-onset-time variance are trending towards significance. Each black dot represents one participant; the white dot represents the average value across participants.

### 2.5 Results studying mean speech measures

The main text focused on coefficient of variation measures. In exploratory, post-hoc analyses, we also studied whether we observed group differences in *mean* speech measures. In particular, we tested whether there were CHR vs. HC group differences in average voice-onset-times, average vowel durations, average speech rates, and, in the diadochokinetic speech tasks, average syllable and intersyllable durations. Past work has found that individuals with and at-risk for psychosis speaker slower than healthy controls, so we might expect to find a similar effect here, as well as evidence that CHR participants produce longer consonants, vowels, syllables, and intersyllable durations. However, we found no evidence CHR vs. HC group differences in speech rate or consonant/vowel/syllable/intersyllable durations. We present these results by speech task.

#### 2.5.1 Diadochokinetic Speech Task

For the diadochokinetic speech tasks, none of the following speech measures differed significantly between the CHR vs. HC groups in either the SMR ("pataka") or AMR ("papapa") subtasks (Figure S14): mean speech rate (AMR: $\beta = 0.13$, s.e. = 0.09, t = 1.48, p = 0.141; SMR: $\beta = 0$, s.e. = 0.06, t = 0.01, p = 0.989), mean voiceless voice-onset-time (AMR: $\beta$ = -0.06, s.e. = 0.08, t = -0.67, p = 0.506; SMR: $\beta$ = -0.01, s.e. = 0.06, t = -0.11, p = 0.91), mean vowel duration (AMR: $\beta$ = -0.14, s.e. = 0.09, t = -1.52, p = 0.133; SMR: $\beta$ = -0.01, s.e. = 0.07, t = -0.19, p = 0.848), mean syllable duration (AMR: $\beta$ = -0.12, s.e. = 0.08, t = -1.4, p = 0.164; SMR: $\beta$ = -0.01, s.e. = 0.06, t = -0.1, p = 0.922), and mean intersyllable duration (AMR: $\beta$ = -0.14, s.e. = 0.11, t = -1.21, p = 0.228; SMR: $\beta$ = 0.03, s.e. = 0.07, t = 0.35, p = 0.727).

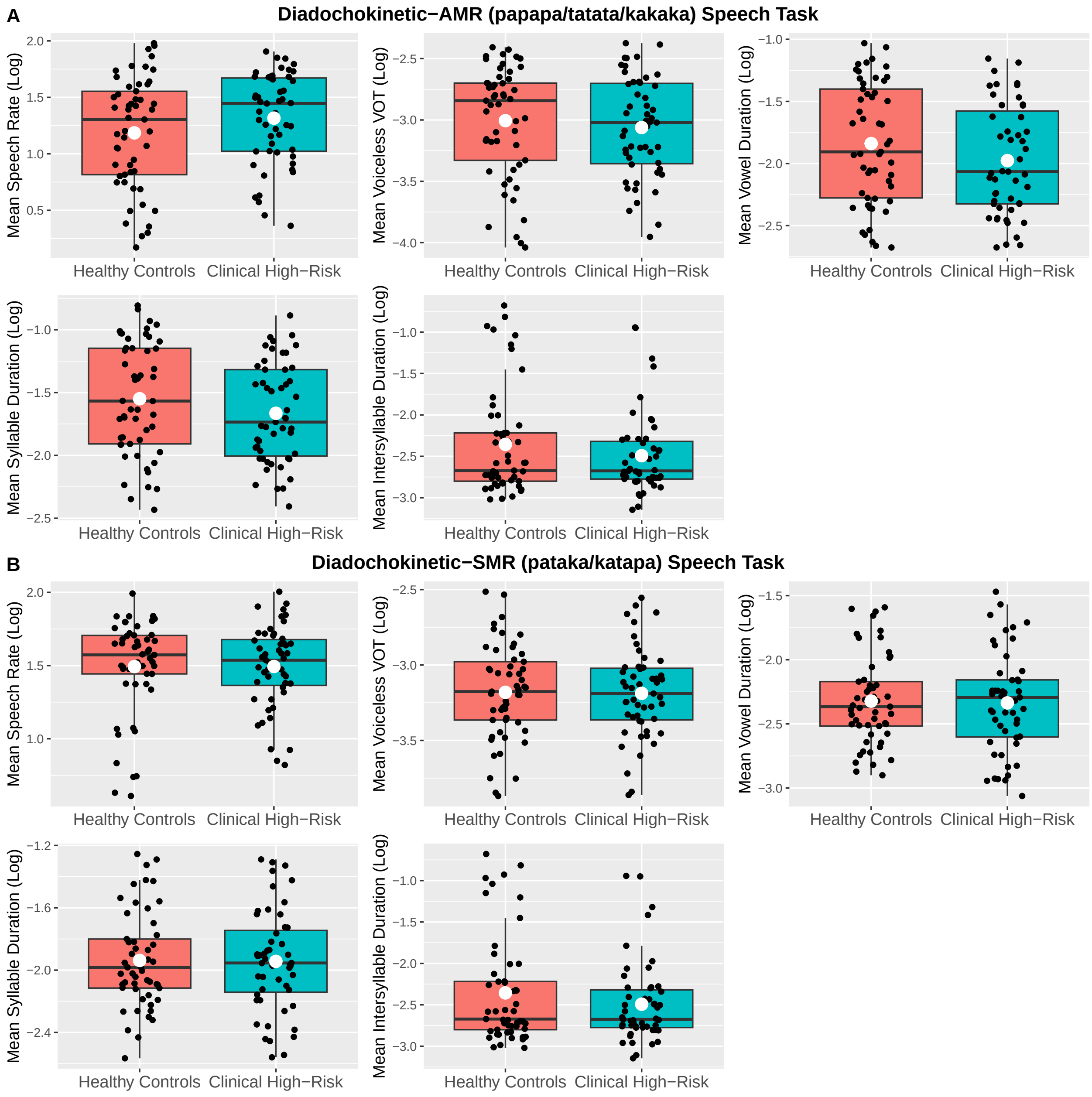


Figure S14: We observed no significant group differences in *mean* speech measure values in either the (A) Diadochokinetic AMR or (B) Diadochokinetic SMR subtasks. Each black dot represents one participant; the white dot represents the average value across participants.

### 2.5.2 Read Speech Task

In the read speech task, none of the following speech measures differed significantly between the CHR vs. HC groups (Figure S15): mean speech rate ($\beta$ = -0.03, s.e. = 0.02, t = -1.23, p = 0.22), mean voiceless voice-onset-time ($\beta$ = 0.01, s.e. = 0.04, t = 0.19, p = 0.853), mean voiced voice-onset-time ($\beta$ = 0.02, s.e. = 0.04, t = 0.44, p = 0.659), and mean vowel duration ($\beta$ = 0.02, s.e. = 0.02, t = 1.02, p = 0.311).

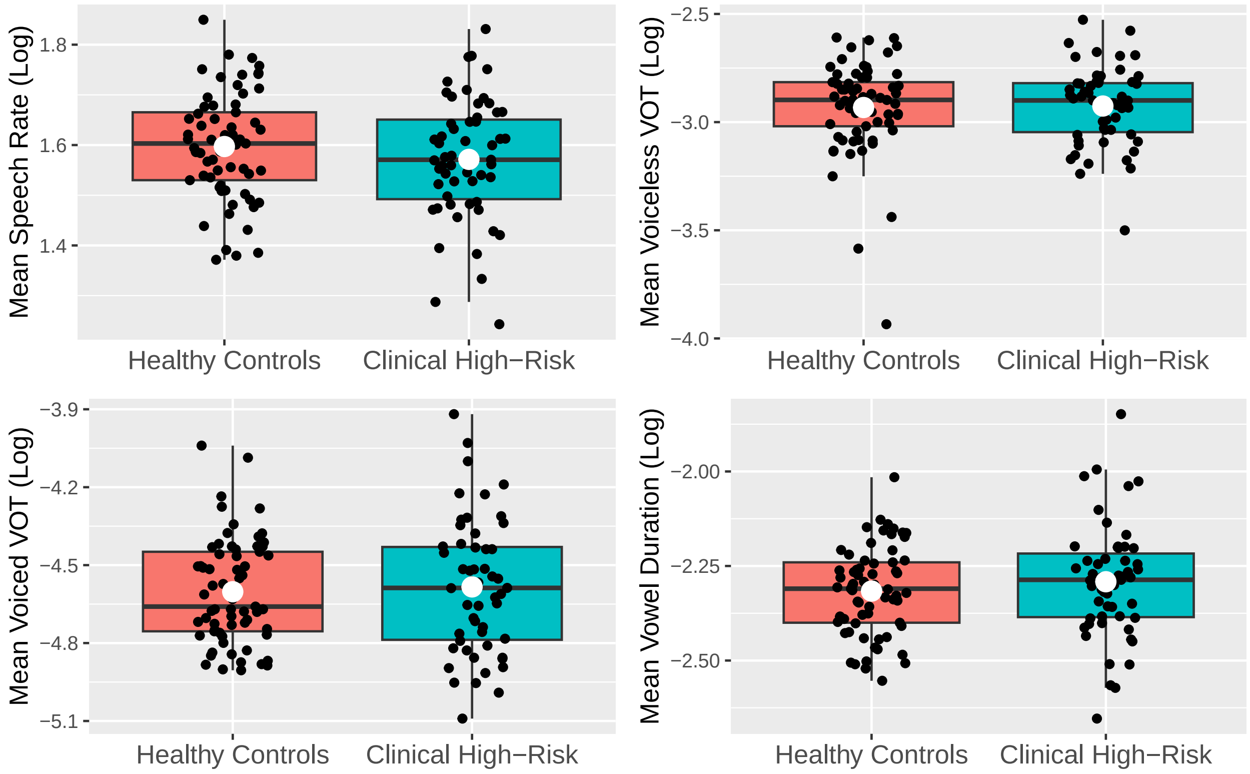


Figure S15: We find no significant CHR vs HC group differences in *mean* speech measures in the reading task. Each black dot represents one participant; the white dot represents the average value across participants.

### 2.5.3 Spontaneous Speech Task

Finally, in the spontaneous speech task, none of the following speech measures differed significantly between the CHR vs. HC groups (Figure S16): mean speech rate ($\beta$ = 0.01, s.e. = 0.03, t = 0.42, p = 0.673), mean voiceless voice-onset-time ($\beta$ = -0.03, s.e. = 0.05, t = -0.55, p = 0.581), mean voiced voice-onset-time ($\beta$ = 0.04, s.e. = 0.06, t = 0.73, p = 0.468), and mean vowel duration ($\beta$ = -0.02, s.e. = 0.03, t = -0.45, p = 0.655).

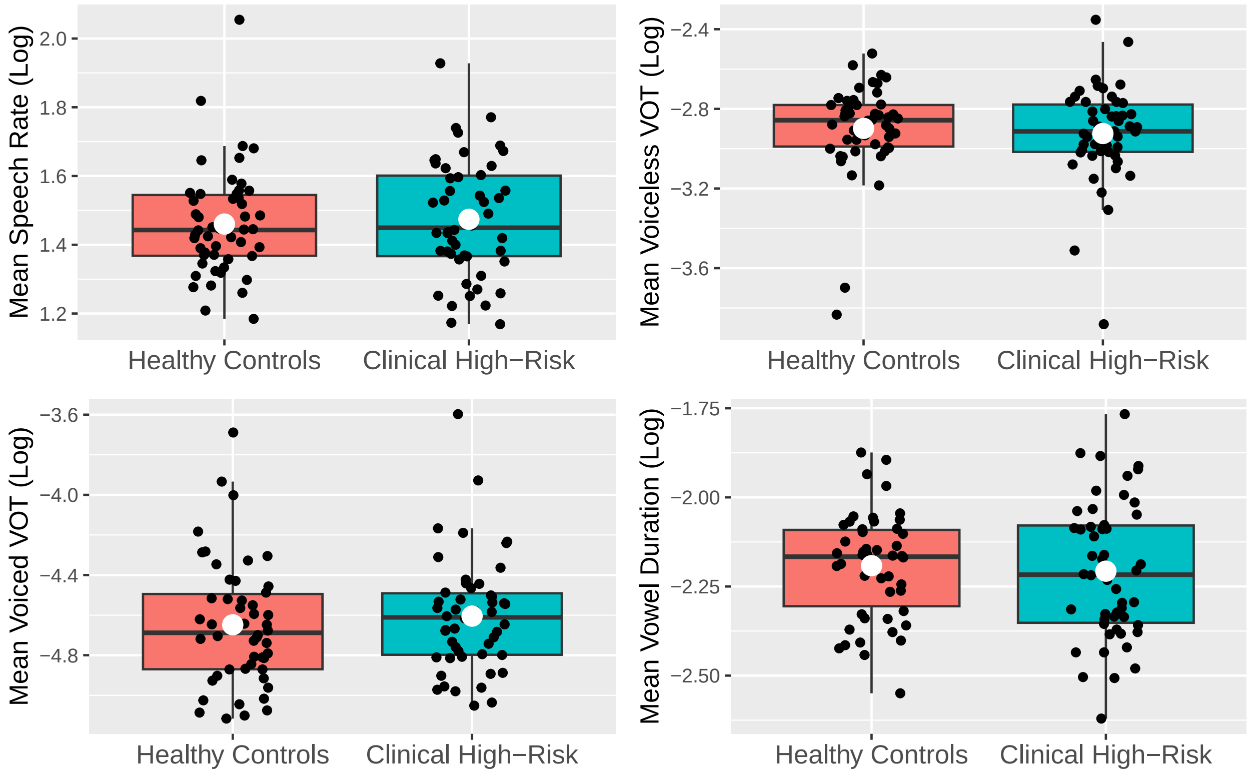


Figure S16: We find no significant CHR vs HC group differences in *mean* speech measures in the spontaneous speech task. Each black dot represents one participant; the white dot represents the average value across participants.

## 2.6 Categorization accuracy results

For each of the speech measures that significantly differed between CHR vs. HC participants, we also run logistic regression models predicting CHR vs. HC status from the acoustic measure to test the categorization accuracy/discriminability of the speech measures. In contrast to previous results, we present these results by speech measure.

### 2.6.1 Voiceless voice-onset-time

We find that the degree of variability in the voice-onset-time of voiceless consonants predicts CHR vs. HC status with 65.38% accuracy in the Diadochokinetic-AMR subtask, 60.58% accuracy in the Diadochokinetic-SMR subtask, and 56.67% accuracy in the read subtask (with a 50% probability cut-off in the logistic regression). We present the full ROC curve (with sensitivity and specificity values) in Figure S17. While the categorization results are considered inadequate (Hosmer et al., 2013; Mandrekar, 2010), we would not expect speech measures on their own to be able to classify CHR vs. HC, so the fact that the measures perform above chance is in itself promising. This suggests that, combined with other measures, speech measures could potentially be useful in identifying individuals at high-risk for psychosis.

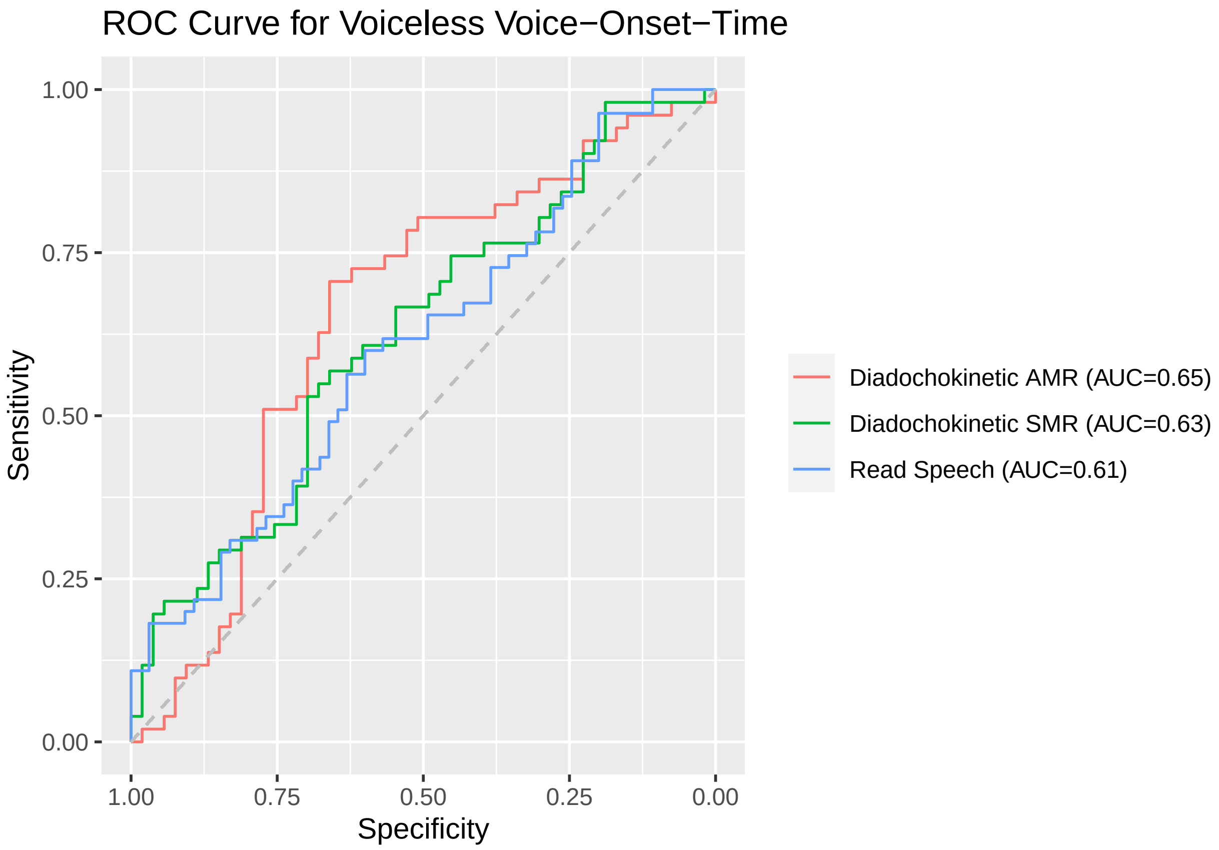


Figure S17: Specificity/sensitivity trade-off for the logistic regression categorization model predicting CHR vs. HC status from the coefficient of variation of voiceless voice-onset-time.

#### 2.6.2 Speech rate

We find that the coefficient of variation in speech rate predicts CHR vs. HC status with 60.58% accuracy in the Diadochokinetic-AMR subtask, 56.73% accuracy in the Diadochokinetic-SMR subtask, and 56.67% accuracy in the read subtask (with a 50% probability cut-off in the logistic regression). We present the full ROC curve (with sensitivity and specificity values) in Figure S18. Again, the fact that the categorization is above chance is promising that speech measures could be helpful in identifying high-risk individuals, in conjunction with other complementary measures.

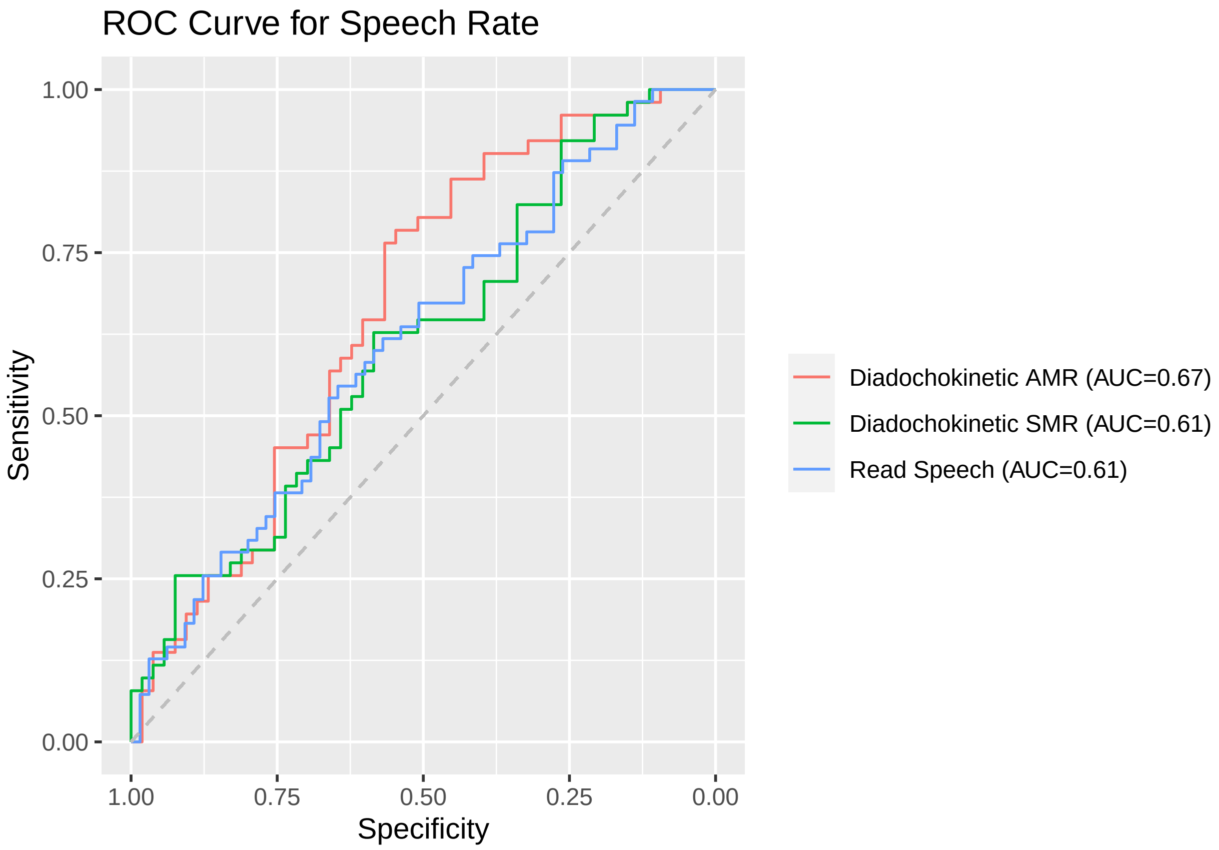


Figure S18: Specificity/sensitivity trade-off for categorization model predicting CHR vs. HC status from the coefficient of variation of speech rate.

### 2.6.3 Combining speech and other measures

Finally, we test how combining speech measures with other motor measures does at categorizing group status. Specifically, we fit a logistic regression model that predicts CHR vs. HC status from a combination of the speech measures that were found to significantly differ by group (voiceless voice-onset-times and speech rate coefficients of variation) and finger-tapping coefficients of variation (dominant hand). We find that this combination of speech and non-speech motor measures predicts group status with 75.32% accuracy in the Diadochokinetic-AMR subtask, 66.23% accuracy in the Diadochokinetic-SMR subtask, and 70.11% accuracy in the read speech task (see Figure S19 for the ROC curve). With the exception of the Diadochokinetic-SMR subtask, this improves over a model that simply uses non-speech motor measures (i.e., finger-tapping coefficients of variation) to predict CHR vs. HC status, which achieves 66.23% accuracy on the Diadochokinetic-AMR subtask, 66.23% accuracy on the Diadochokinetic-SMR subtask, and 64.37% accuracy on the read speech task. Overall, this suggests that, when combined with other clinical and motor measures, speech measures has diagnostic value and this should be further studied in the future.

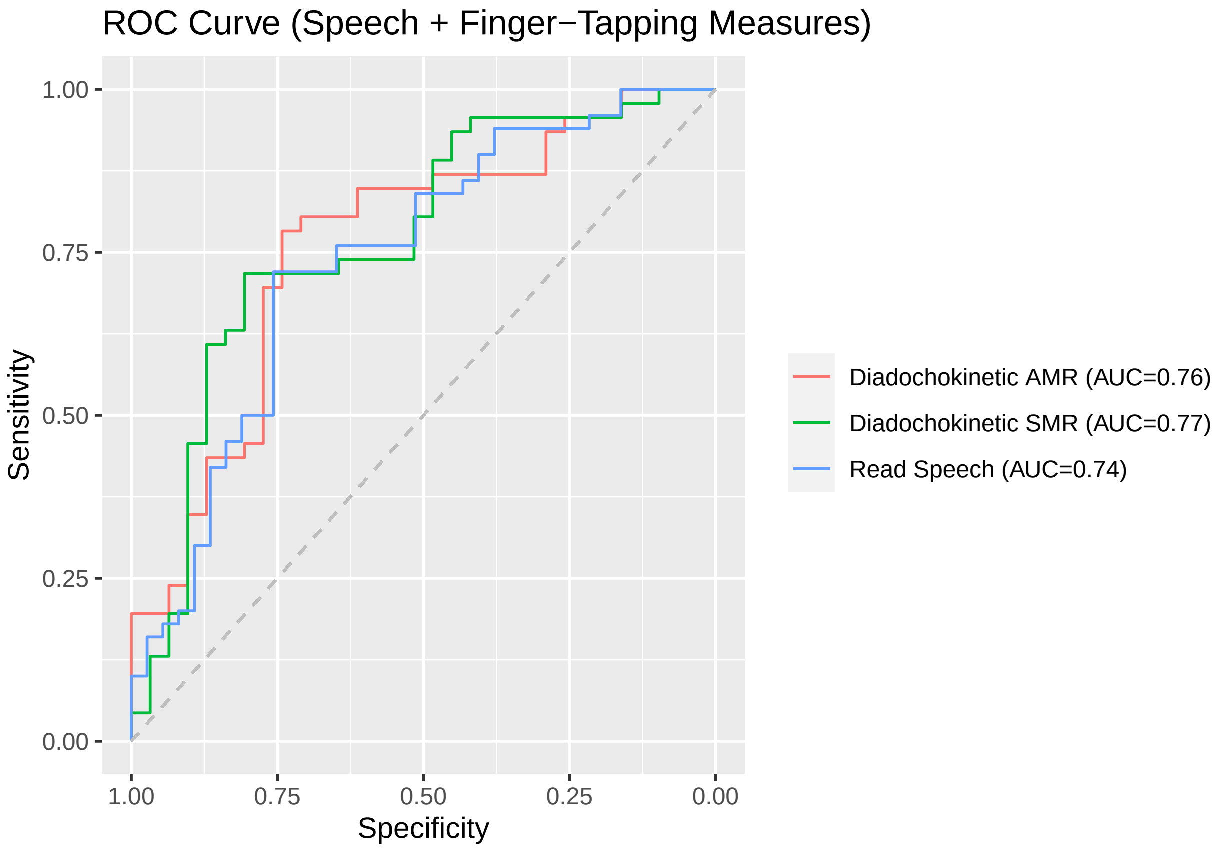


Figure S19: Specificity/sensitivity trade-off for categorization model predicting CHR vs. HC status from both speech measures and non-speech motor measures (finger-tapping coefficient of variation in the dominant hand).

## 2.7 Results comparing the in-person and remote subgroups

Due to space constraints, the main text only presented some of the graphs comparing the subset of participants tested in-person prior to the pandemic and the subset of participants tested remotely during the pandemic. Here, we present the full set of results, in which we often observe quite large CHR vs. HC group differences in the In-Person subgroup and **reduced** group differences in the Remote subgroup. For each of the two speech measures that showed significant group differences overall, we tested whether they also showed significant group differences within both the in-person and remote subsets across the three tasks that showed significant group effects (i.e., Diadochokinetic-AMR, Diadochokinetic-SMR, and Read speech tasks).

For variability in voiceless voice-onset-time (Figure S20), we observe significant CHR vs. HC group differences in the in-person subgroup (Diadochokinetic-AMR: $\beta = 0.17$, s.e. $= 0.07$, t $= 2.52$, p $= 0.015$; Diadochokinetic-SMR: $\beta = 0.2$, s.e. $= 0.07$, t $= 2.83$, p $= 0.007$; Read: $\beta = 0.15$, s.e. $= 0.05$, t $= 2.91$, p $= 0.005$), but not the remote subgroup (Diadochokinetic-AMR: $\beta = 0.07$, s.e. $= 0.07$, t $= 1.05$, p $= 0.297$; Diadochokinetic-SMR: $\beta = 0.02$, s.e. $= 0.06$, t $= 0.25$, p $= 0.807$; Read: $\beta = 0.06$, s.e. $= 0.05$, t $= 1.17$, p $= 0.248$), though we note that the in-person and remote subgroups show similar qualitative patterns in the Read Speech task.

For variability in speech rate (Figure S21), except for the Diadochokinetic-SMR speech task, where we observe significant group differences in the in-person subgroup ($\beta = 0.47$, s.e. $= 0.16$, t $= 2.89$, p $= 0.006$), but not the remote subgroup ($\beta = 0$, s.e. $= 0.12$, t $= 0.03$, p $= 0.976$), results are qualitatively much more similar across the in-person and remote subgroups. Nonetheless, we do observe statistical differences between groups in the other two speech tasks. In particular, we observe statistically significant group differences in the remote subgroup (Diadochokinetic-AMR: $\beta = 0.43$, s.e. $= 0.16$, t $= 2.67$, p $= 0.01$; Read: $\beta = 0.16$, s.e. $= 0.07$, t $=$

2.18, p = 0.034), but not the in-person subgroup (Diadochokinetic-AMR: $\beta = 0.36$, s.e. = 0.18, t = 2, p = 0.051; Read: $\beta = 0.11$, s.e. = 0.07, t = 1.52, p = 0.135). However, based on the plots and the fact that results approach significance, this difference is likely due to the loss of power that results from cutting the sample in half.

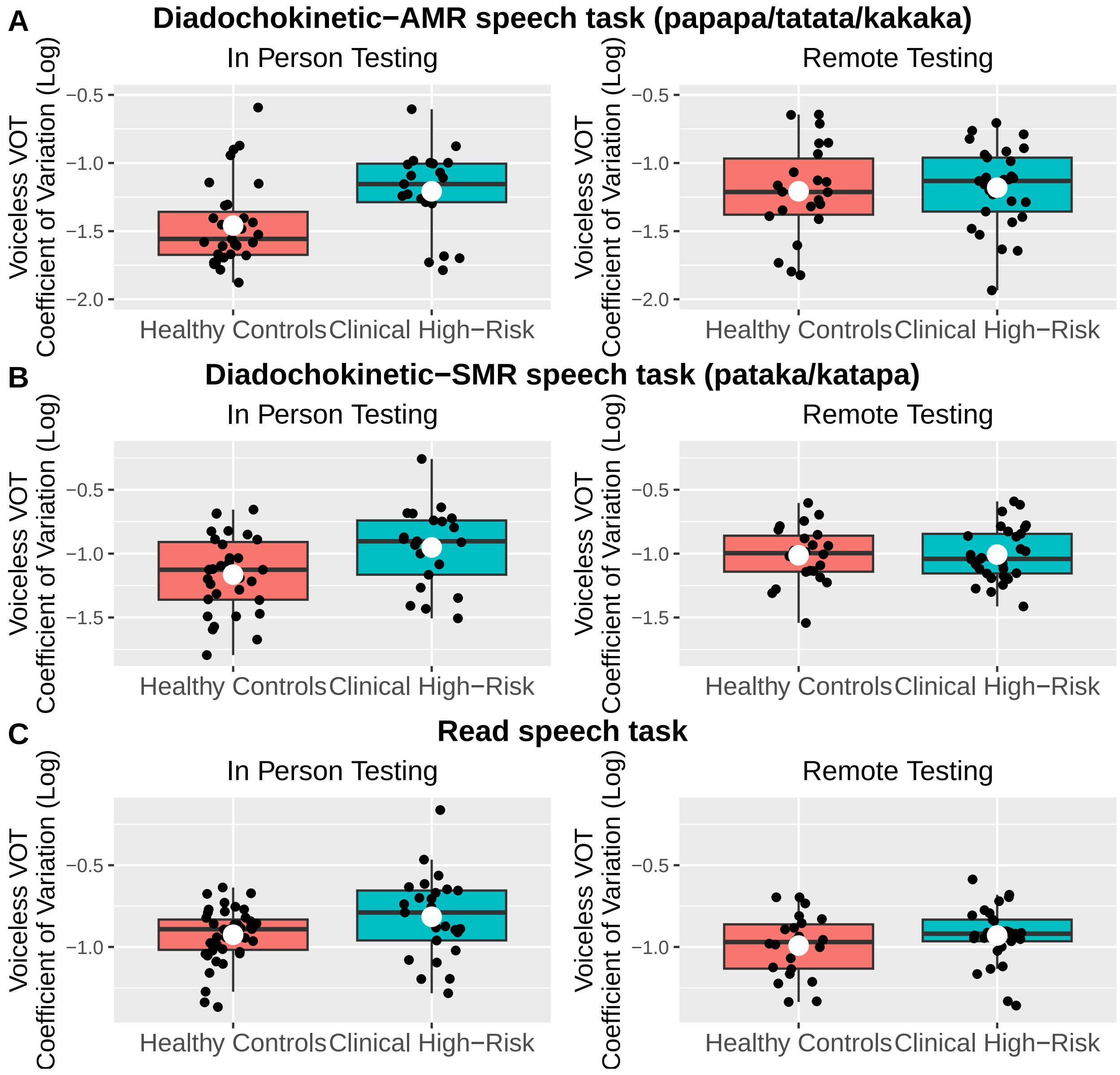


Figure S20: Comparison of variability in voiceless voice onset time between Clinical High-Risk (left bar in each subplot) and Healthy Control participants (right bar in each subplot), broken down by whether the participants were recorded in-person prior to the pandemic (left plot in each row) or remotely during (right plot in each row). (A) shows results for the Diadochokinetic-AMR (papapa/tatata/kakaka) speech task; (B) shows results for the Diadochokinetic-SMR (pataka/katapa) speech task; and (C) shows results for the Read speech task. Each black dot represents one participant; the white dot represents the average value across participants. Overall, we observe that starker group differences in the in-person subgroup, relative to the remote subgroup.

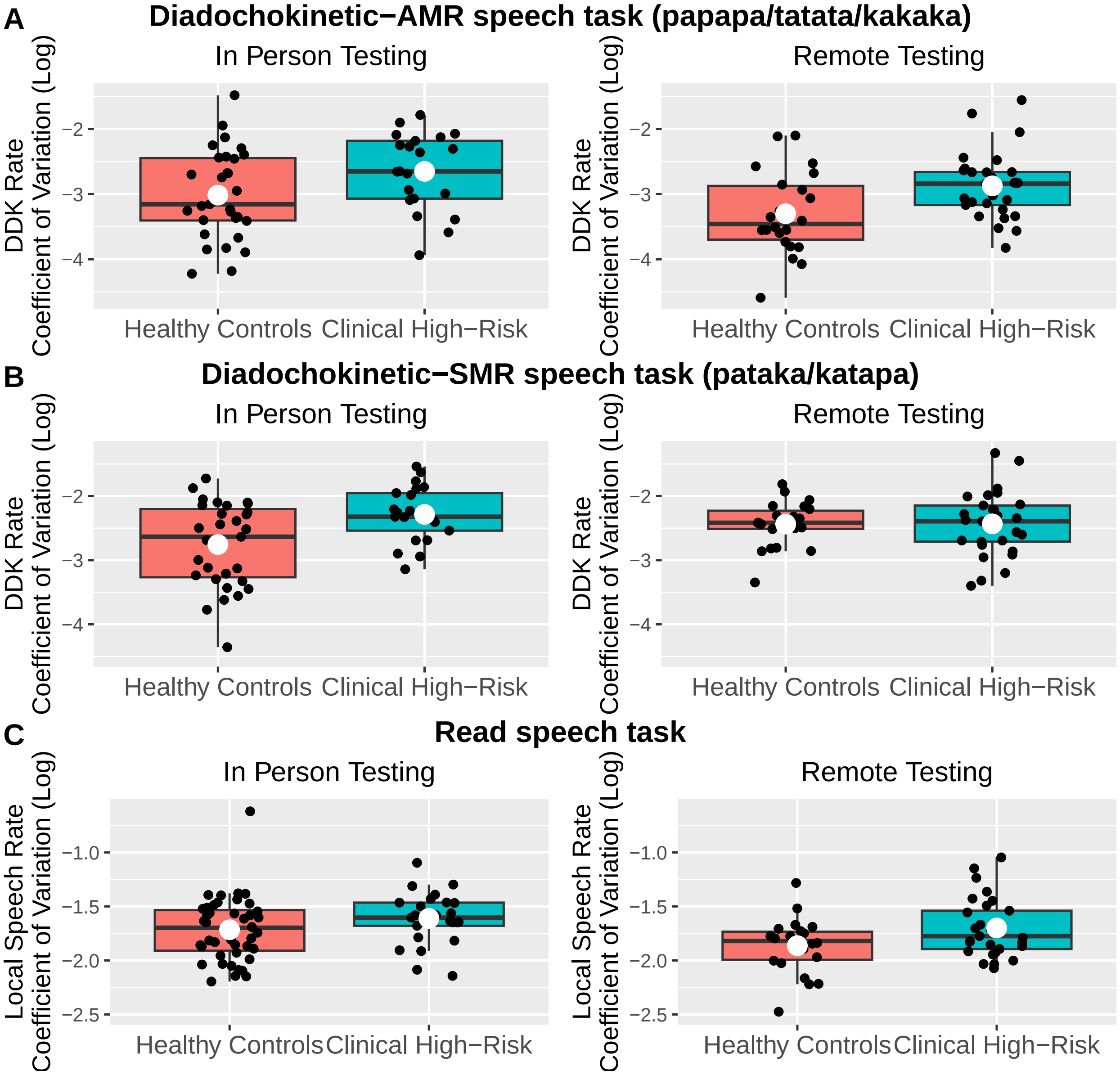


Figure S21: Comparison of variability in speech rate between Clinical High-Risk (left bar in each subplot) and Healthy Control participants (right bar in each subplot), broken down by whether the participants were recorded in-person prior to the pandemic (left plot in each row) or remotely during (right plot in each row). (A) shows results for the Diadochokinetic-AMR (papapa/tatata/kakaka) speech task; (B) shows results for the Diadochokinetic-SMR (pataka/katapa) speech task; and (C) shows results for the Read speech task. Each black dot represents one participant; the white dot represents the average value across participants. Overall, compared to the variability in consonant production, results are qualitatively more similar across the in-person and remote groups (with the exception of the Diadochokinetic-SMR subtask, where we again observe a starker group difference in the in-person subgroup, relative to the remote one).

## 2.8 Analyses studying the relationship between speech measures and demographics

Past work has found that some measures intended to measure speech/language differences actually tapped into sociodemographic factors. We tested whether this explained the group differences in speech variability, by

studying the relationship between the speech measures and all sociodemographic factors reported in Table 3 of the main text (age, sex, race, ethnicity (Hispanic vs. not), and first language), within the Diadochokinetic-AMR, SMR, and Read speech tasks (i.e., those that showed significant group differences). To do so, for each sociodemographic factor, for each subtask, for each speech measure, we fit a linear regression model predicting the speech measure from two predictors: the sociodemographic factor and group (CHR vs HC), without interactions. When studying variability in voiceless stop production, we additionally included a third predictor, average speech rate, as a control. Our results suggest that sociodemographics cannot explain the group differences in speech measure variability observed in the main paper.

### 2.8.1 Age

We found no consistent relationship between our speech measures and participant age (Figure S22). While we did find a significant relationship between age and variability in voiceless stop production AMR subtask ($\beta$ = 0.02, s.e. = 0.01, t = 2.45, p = 0.016), such that older participants produced more variable speech, we found no such evidence in the SMR ($\beta$ = -0.01, s.e. = 0.01, t = -1.76, p = 0.081) or Read ($\beta$ = 0, s.e. = 0.01, t = -0.53, p = 0.597) subtasks. In addition, we found no relationship between age and variability in speech rate in any of the AMR ($\beta$ = -0.01, s.e. = 0.02, t = -0.52, p = 0.605), SMR ($\beta$ = -0.02, s.e. = 0.02, t = -1.37, p = 0.174), or Read ($\beta$ = 0.01, s.e. = 0.01, t = 0.6, p = 0.547) speech tasks.

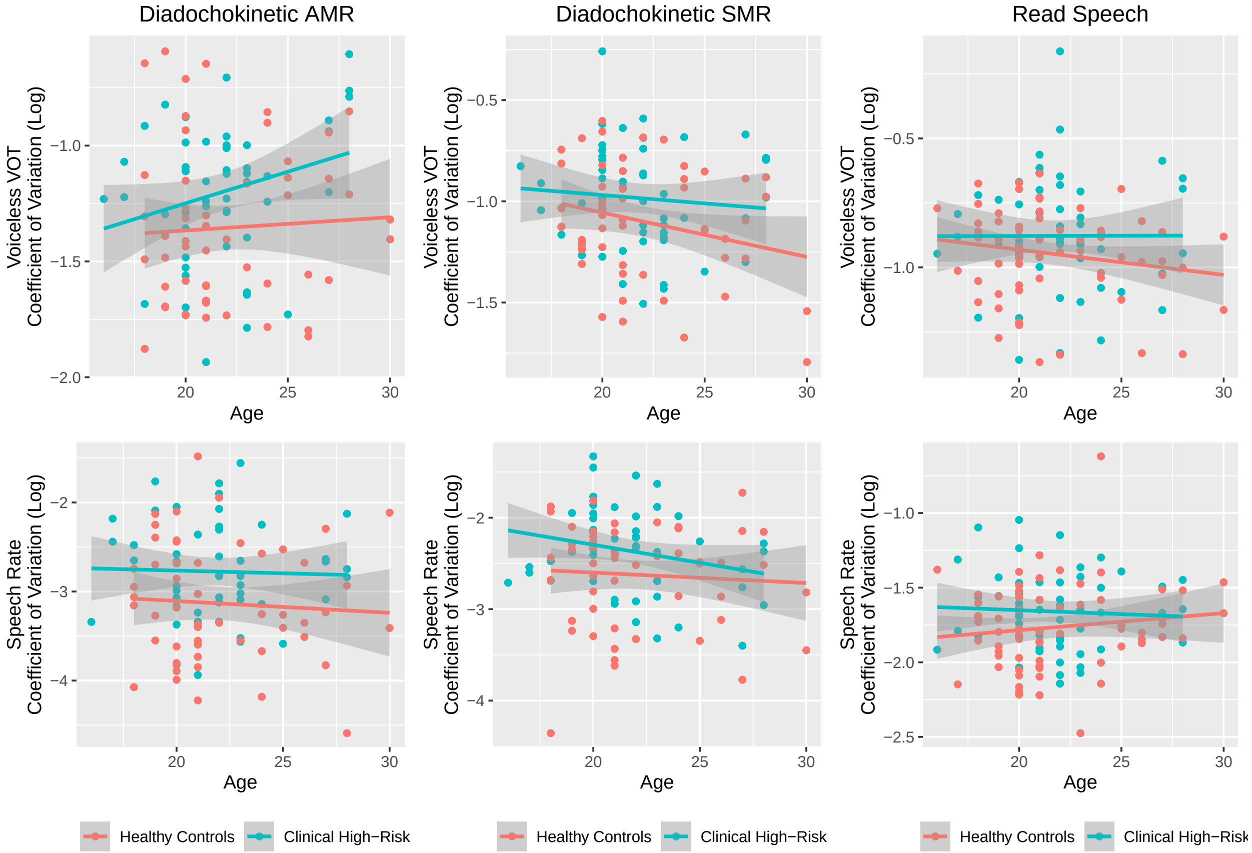


Figure S22: We found no consistent relationship between speech measures and age in our sample. The top row shows results for the variability in voiceless consonant production measure, while the bottom row shows results for the variability in speech rate measure. The leftmost column corresponds to the Diadochokinetic-AMR (papapa/tatata/kakaka) speech task; the middle column corresponds to the Diadochokinetic-SMR (pataka/katapa) speech task; the rightmost column corresponds to the Read speech task. Each point represents one participant; the lines represent the lines of best-fit by participant group (CHR vs HC), with shaded regions showing standard errors of the regression fit.

### 2.8.2 Sex

We found no relationship between participant sex and speech measures in our sample (Figure S23). In particular, participant sex was unrelated to both variability in voiceless stop production (Diadochokinetic-AMR: $\beta$ = 0.02, s.e. = 0.05, t = 0.38, p = 0.706; Diadochokinetic-SMR: $\beta$ = 0, s.e. = 0.05, t = 0.03, p = 0.979; Read: $\beta$ = 0.03, s.e. = 0.04, t = 0.86, p = 0.394) and variability in speech rate (Diadochokinetic-AMR: $\beta$ = 0.05, s.e. = 0.12, t = 0.42, p = 0.673; Diadochokinetic-SMR: $\beta$ = -0.06, s.e. = 0.1, t = -0.61, p = 0.541; Read: $\beta$ = 0.1, s.e. = 0.05, t = 1.93, p = 0.056).

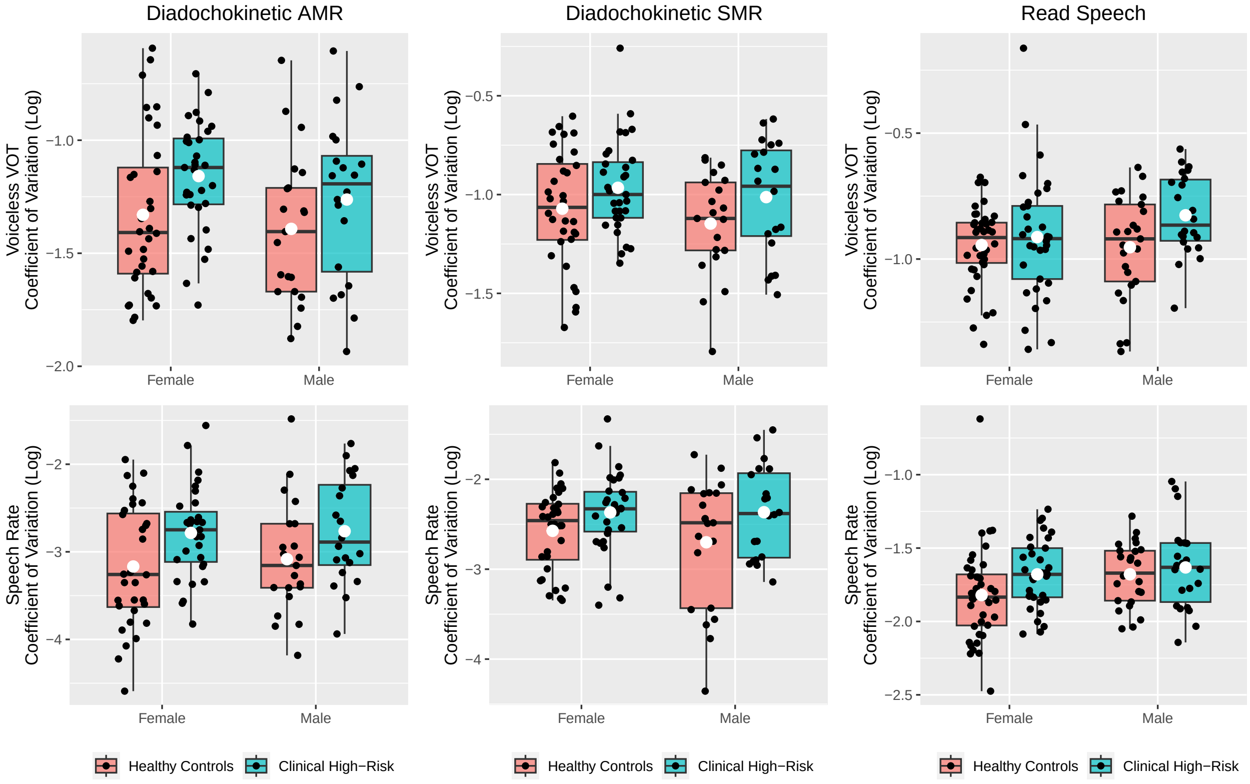


Figure S23: We found no relationship between participant sex and speech measures in our sample. The top row shows results for the variability in voiceless consonant production measure, while the bottom row shows results for the variability in speech rate measure. The leftmost column corresponds to the Diadochokinetic-AMR (papapa/tatata/kakaka) speech task; the middle column corresponds to the Diadochokinetic-SMR (pataka/katapa) speech task; the rightmost column corresponds to the Read speech task. Each black dot represents one participant; the white dot represents the average value across participants.

### 2.8.3 Race

To study the potential role of racial identity, we focused our analyses on pairwise comparisons between (i) individuals who self-identified as Black, (ii) individuals who self-identified as Asian, and (iii) individuals who self-identified as white. This was done to ensure sufficiently-sized comparison groups. Even with this decision, our conclusions are still limited by sample size (e.g., some N's near 10), and future work should follow-up on this question using large enough samples that allow for fully-powered, and more nuanced, analyses.

Results are visually presented in Figure S24. Across all three tasks and all pairwise comparisons, we find no relationship between racial identity and variability in speech measures. In particular, within the pairwise comparison of Asian and white participants, we find no relationship between racial identity and either variability in voiceless voice-onset-times (AMR: $\beta$ = 0.04, s.e. = 0.06, t = 0.59, p = 0.558, SMR: $\beta$ = 0.07, s.e. = 0.07, t = 1.11, p = 0.272, Read: $\beta$ = 0.03, s.e. = 0.04, t = 0.64, p = 0.527) or variability in speech

rate (AMR: $\beta$ = -0.07, s.e. = 0.16, t = -0.46, p = 0.644, SMR: $\beta$ = -0.03, s.e. = 0.14, t = -0.24, p = 0.81, Read: $\beta$ = 0.04, s.e. = 0.06, t = 0.61, p = 0.543).

Similarly, within the pairwise comparison of Asian and Black participants, we find no relationship between racial identity and either variability in voiceless VOT (AMR: $\beta$ = -0.04, s.e. = 0.08, t = -0.58, p = 0.562, SMR: $\beta$ = 0.04, s.e. = 0.09, t = 0.52, p = 0.609, Read: $\beta$ = 0.06, s.e. = 0.07, t = 0.85, p = 0.4) or variability in speech rate (AMR: $\beta$ = -0.16, s.e. = 0.2, t = -0.8, p = 0.427, SMR: $\beta$ = -0.04, s.e. = 0.18, t = -0.22, p = 0.826, Read: $\beta$ = -0.06, s.e. = 0.08, t = -0.75, p = 0.456).

Finally, within the pairwise comparison of Black and white participants, we again find no relationship between racial identity and either variability in voiceless VOT (AMR: $\beta$ = -0.1, s.e. = 0.07, t = -1.43, p = 0.159, SMR: $\beta$ = -0.05, s.e. = 0.07, t = -0.66, p = 0.51, Read: $\beta$ = 0.06, s.e. = 0.05, t = 1.15, p = 0.254) or variability in speech rate (AMR: $\beta$ = -0.21, s.e. = 0.17, t = -1.19, p = 0.24, SMR: $\beta$ = 0.02, s.e. = 0.16, t = 0.12, p = 0.902, Read: $\beta$ = -0.13, s.e. = 0.09, t = -1.45, p = 0.15).

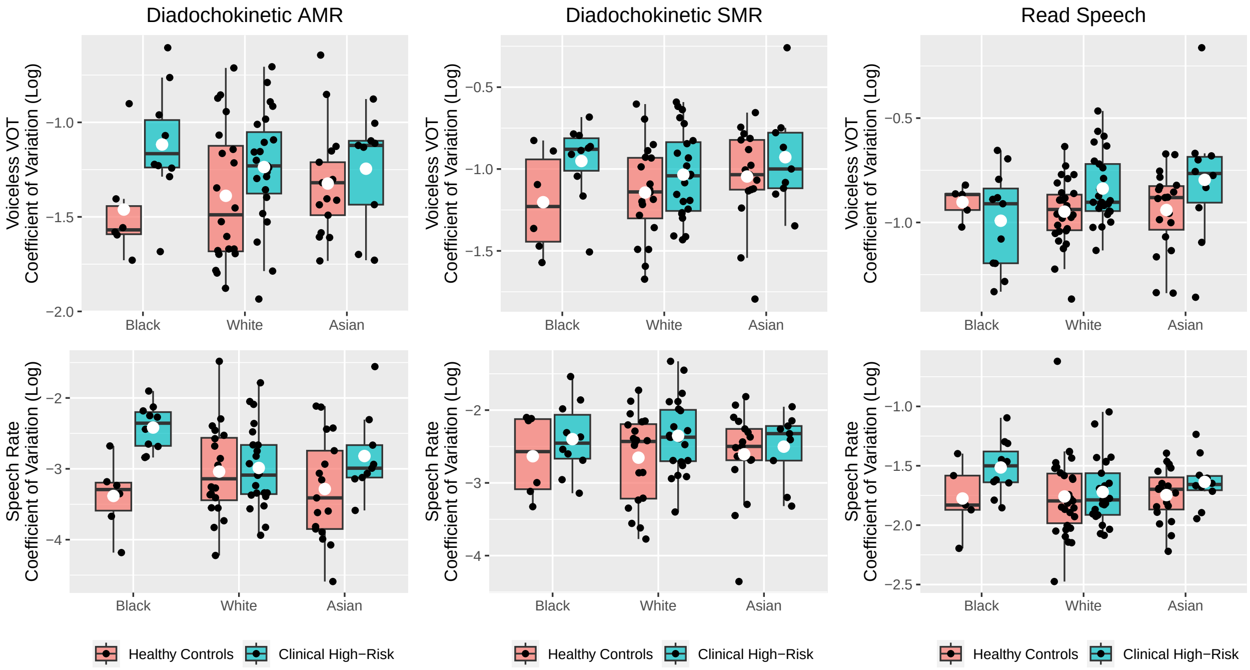


Figure S24: We observed no relationship between racial identity and speech variability in our sample. The top row shows results for the variability in voiceless consonant production measure, while the bottom row shows results for the variability in speech rate measure. The leftmost column corresponds to the Diadochokinetic-AMR (papapa/tatata/kakaka) speech task; the middle column corresponds to the Diadochokinetic-SMR (pataka/katapa) speech task; the rightmost column corresponds to the Read speech task. Each black dot is one participant; the white dot is the average across participants.

### 2.8.4 Ethnicity (Hispanic)

We found no relationship between participant ethnicity (focusing on Hispanic vs Non-Hispanic individuals; Figure S25) and variability in speech measures. In particular, participant ethnicity was unrelated to both variability in voiceless stop production (Diadochokinetic-AMR: $\beta$ = -0.03, s.e. = 0.06, t = -0.48, p = 0.635; Diadochokinetic-SMR: $\beta$ = -0.06, s.e. = 0.06, t = -0.99, p = 0.325; Read: $\beta$ = -0.01, s.e. = 0.05, t = -0.2, p = 0.841) and variability in speech rate (Diadochokinetic-AMR: $\beta$ = 0.16, s.e. = 0.16, t = 1.01, p = 0.317; Diadochokinetic-SMR: $\beta$ = 0.2, s.e. = 0.13, t = 1.5, p = 0.136; Read: $\beta$ = -0.05, s.e. = 0.07, t = -0.72, p = 0.475).

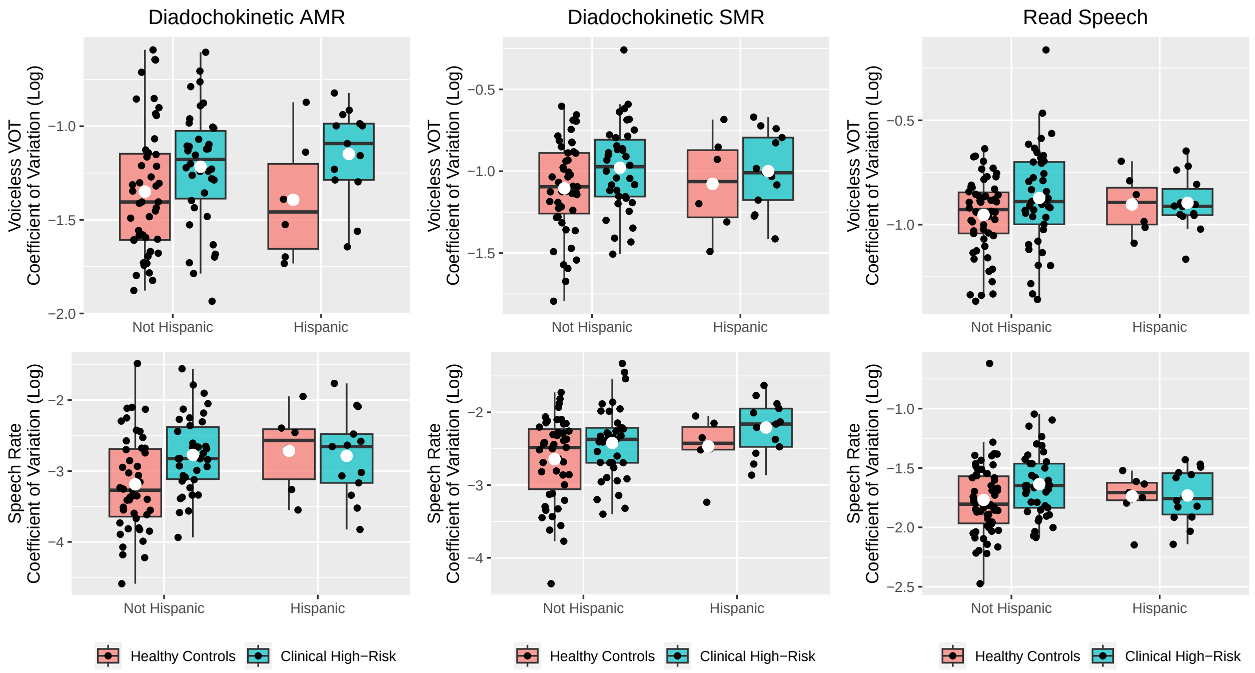


Figure S25: We found no relationship between participant ethnicity (Hispanic vs. Not Hispanic) and speech measures in our sample. The top row shows results for the variability in voiceless consonant production measure, while the bottom row shows results for the variability in speech rate measure. The leftmost column corresponds to the Diadochokinetic-AMR (papapa/tatata/kakaka) speech task; the middle column corresponds to the Diadochokinetic-SMR (pataka/katapa) speech task; the rightmost column corresponds to the Read speech task. Each black dot is one participant; the white dot is the average across participants.

### 2.8.5 First Language

We found no relationship between language status (participants who reported English to be their first language vs. participants who did not) and variability in speech measures (Figure S26). In particular, first language status did not relate to either variability in voiceless stop production (Diadochokinetic-AMR: $\beta$ = -0.03, s.e. = 0.06, t = -0.55, p = 0.583; Diadochokinetic-SMR: $\beta$ = -0.04, s.e. = 0.06, t = -0.71, p = 0.482; Read: $\beta$ = -0.01, s.e. = 0.05, t = -0.19, p = 0.853) or variability in speech rate (Diadochokinetic-AMR: $\beta$ = 0.21, s.e. = 0.16, t = 1.29, p = 0.201; Diadochokinetic-SMR: $\beta$ = 0.11, s.e. = 0.13, t = 0.85, p = 0.398; Read: $\beta$ = 0.08, s.e. = 0.07, t = 1.19, p = 0.237).

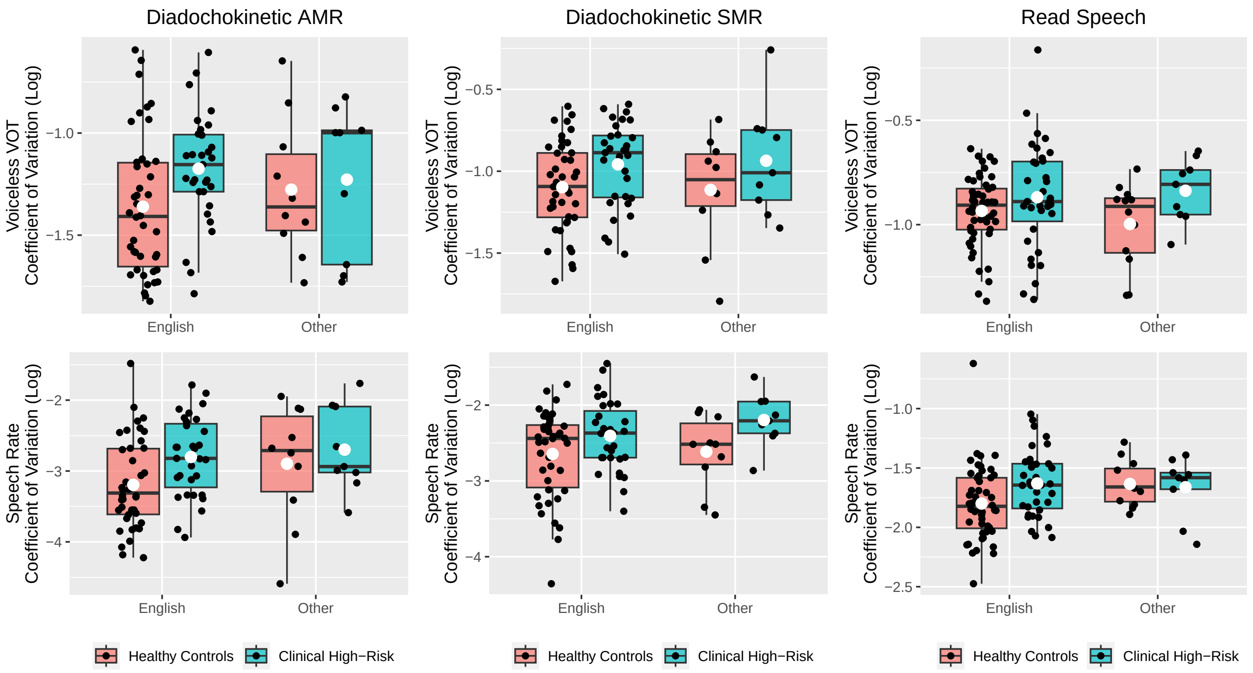


Figure S26: We found no relationship between participants' first language (English vs. Other) and speech measure variability. The top row shows results for the variability in voiceless consonant production measure, while the bottom row shows results for the variability in speech rate measure. The leftmost column corresponds to the Diadochokinetic-AMR (papapa/tatata/kakaka) speech task; the middle column corresponds to the Diadochokinetic-SMR (pataka/katapa) speech task; the rightmost column corresponds to the Read speech task. Each black dot is one participant; the white dot is the average across participants.

## 2.9 Analyses studying the relationship between non-speech motor measures vs. symptom severity and risk of conversion

Past work has established that motor symptoms (e.g., finger tapping scores) correlate with symptom severity and risk of conversion scores in individuals at clinical high-risk for psychosis. To better understand why we did not observe correlations between speech measures and our validation measures, we checked for these relationships in our sample. Surprisingly, we found no correlations between finger tapping and symptom severity and risk of conversion scores (Figure S27).

In particular, we found that the coefficient of variation of finger tapping in the dominant hand did not correlate with the SIPS Positive Total score ($\beta$ = -0.19, s.e. = 0.7, t = -0.27, p = 0.786), SIPS Negative Total score ($\beta$ = -0.91, s.e. = 1.12, t = -0.81, p = 0.421), SIPS Disorganized Total score ($\beta$ = 0.04, s.e. = 0.51, t = 0.08, p = 0.935), SIPS G3 "Motor Difficulties" score ($\beta$ = 0.18, s.e. = 0.22, t = 0.83, p = 0.409), or the SIPS-RC Risk of Conversion score ($\beta$ = 0.07, s.e. = 0.46, t = 0.15, p = 0.884). Similarly, we found no correlation between the coefficient of variation of finger tapping in the non-dominant hand and the SIPS Positive Total score ($\beta$ = -0.06, s.e. = 0.72, t = -0.08, p = 0.938), SIPS Negative Total score ($\beta$ = -1.86, s.e. = 1.21, t = -1.54, p = 0.13), SIPS Disorganized Total score ($\beta$ = 0.27, s.e. = 0.55, t = 0.49, p = 0.626), SIPS G3 "Motor Difficulties" score ($\beta$ = -0.06, s.e. = 0.23, t = -0.24, p = 0.813), or the SIPS-RC Risk of Conversion score ($\beta$ = 0.4, s.e. = 0.48, t = 0.84, p = 0.403). These results suggest that our sample did not show the typical motor profile, that we may not have sufficient power to identify correlations with clinical measures, and/or that our convergent non-speech motor measure was affected by the shift to remote testing.

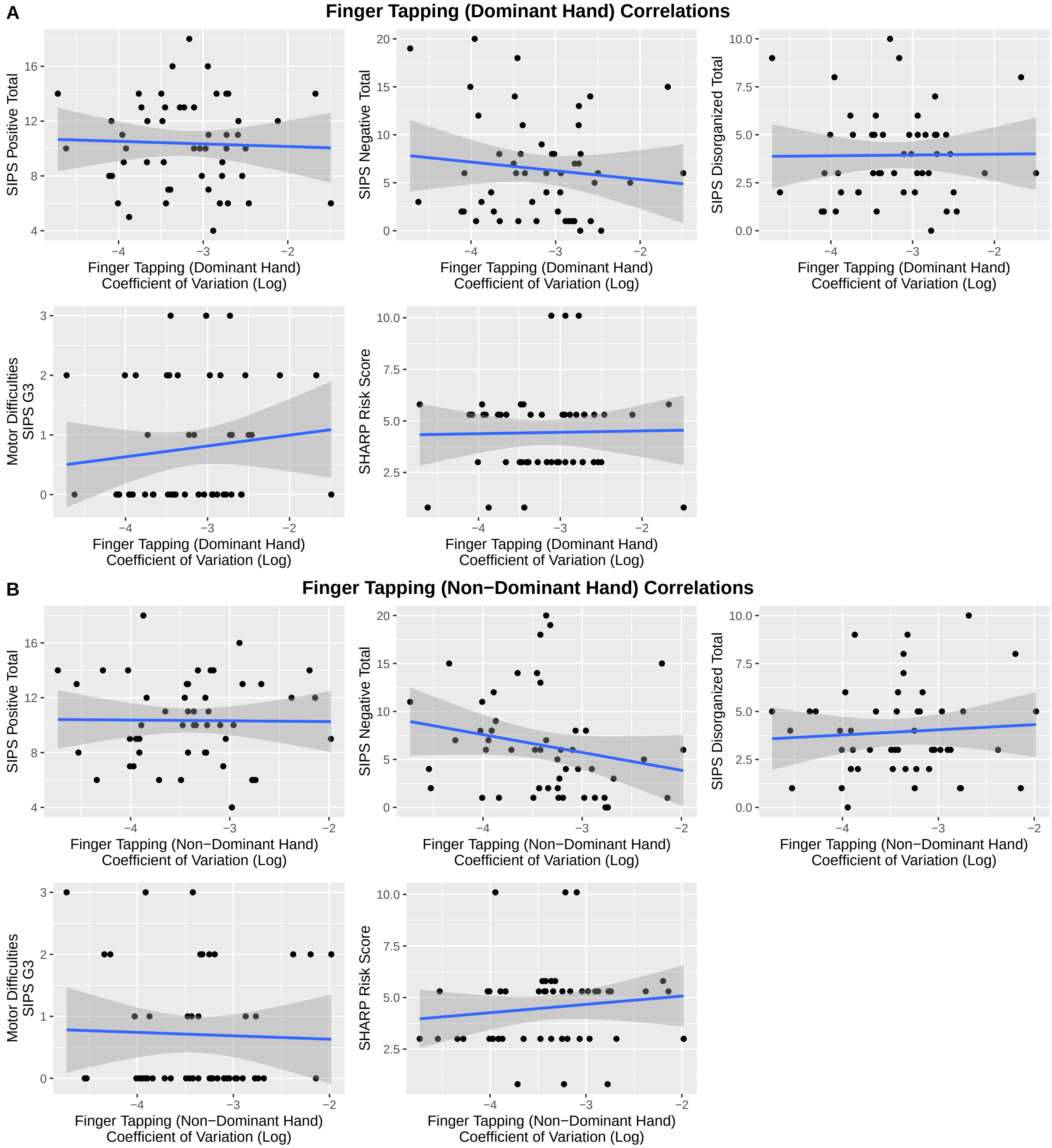


Figure S27: Post-hoc analyses revealed that, contrary to past findings, non-speech motor symptoms, both as measured by (A) the coefficient of variation in finger tapping in the dominant hand and (B) the coefficient of variation in finger tapping in the non-dominant hand, did not correlate with symptom severity or risk of conversion scores in our sample of individuals at clinical high-risk for psychosis.

## 2.10 Analyses with a subset of participants removed

We reconducted our main analyses with two CHR participants removed: one was later identified as being in remission and another had more than 7mo elapse between their clinical interview and speech tasks.

#### 2.10.1 Clinical High-Risk vs. Healthy Control group differences

We found qualitatively similar, though statistically different, group difference results to those reported in the main text. We found that the coefficient of variation of voiceless consonants significantly predicted CHR vs HC in the Diadochokinetic SMR ("pataka"/"katapa") speech samples, but was just above the significance threshold in the Diadochokinetic AMR ("papapa"/"tataka"/"kakaka") and Read speech samples. We found that the coefficient of variation of speech rate still significantly predicted group status (CHR vs. HC) in the Diadochokinetic AMR and Diadochokinetic SMR speech samples, but was just above the significance threshold in the Read speech samples. Finally, we additionally found that the coefficient of variation in intersyllable duration significantly predicted CHR vs HC in the Diadochokinetic SMR speech samples, but not the Diadochokinetic SMR speech samples. Because of the large number of graphs and their similarity to those reported in the main text, we just provide details on the statistical analyses in Table S2.

Table S 2: Summary of Clinical High-Risk vs. Healthy Control group difference analysis outcomes with two clinical-high-risk participants excluded. Bold denotes a significant difference ($p<0.05$); italics denotes a trending difference ($p<0.10$).

| Measure | Subtask | Beta | s.e. | z | p |
|---|---|---|---|---|---|
| Consonant Production Measures | | | | | |
| CoVs in Voiceless VOTs | *AMR* | *-1.64* | *0.86* | *-1.91* | *0.06* |
| | **SMR** | **-2.04** | **0.89** | **-2.29** | **0.02** |
| | *Read* | *-2.04* | *1.05* | *-1.95* | *0.05* |
| | PB&J | 0.58 | 0.99 | 0.59 | 0.56 |
| CoVs in Voiced VOTs | AMR | n/a | n/a | n/a | n/a |
| | SMR | n/a | n/a | n/a | n/a |
| | Read | 0.3 | 0.51 | 0.58 | 0.56 |
| | PB&J | -0.54 | 0.4 | -1.36 | 0.17 |
| Speech Rate Measures | | | | | |
| CoVs in Speech Rate | **AMR** | **-0.93** | **0.35** | **-2.69** | **0.01** |
| | **SMR** | **-0.94** | **0.41** | **-2.28** | **0.02** |
| | *Read* | *-1.37* | *0.71* | *-1.93* | *0.05* |
| | PB&J | -0.24 | 0.41 | -0.59 | 0.55 |
| Vowel Production Measures | | | | | |
| CoVs in Vowel Duration | AMR | -0.76 | 0.52 | -1.47 | 0.14 |
| | SMR | -0.76 | 0.56 | -1.36 | 0.17 |
| | Read | -1.94 | 1.96 | -0.99 | 0.32 |
| | PB&J | -0.12 | 1.5 | -0.08 | 0.94 |
| Formant Dispersion (20%) | *AMR* | *-0.76* | *0.44* | *-1.74* | *0.08* |
| | SMR | -0.71 | 0.52 | -1.36 | 0.17 |
| | Read | -0.63 | 1.36 | -0.46 | 0.64 |
| | PB&J | 0.79 | 1.39 | 0.57 | 0.57 |

Table S 2: Summary of Clinical High-Risk vs. Healthy Control group difference analysis outcomes with two clinical-high-risk participants excluded. Bold denotes a significant difference (p<0.05); italics denotes a trending difference (p<0.10).

| Measure | Subtask | Beta | s.e. | z | p |
|---|---|---|---|---|---|
| Change in Formant Dispersion (20/50) | AMR | -0.01 | 0.01 | -0.56 | 0.58 |
| | SMR | 0 | 0.01 | -0.32 | 0.75 |
| | Read | 0 | 0.01 | -0.15 | 0.88 |
| | PB&J | 0.01 | 0.01 | 0.66 | 0.51 |
| Vowel Overlap (Phonetic Competition) | AMR | n/a | n/a | n/a | n/a |
| | SMR | n/a | n/a | n/a | n/a |
| | Read | -0.07 | 0.14 | -0.52 | 0.61 |
| | PB&J | -0.31 | 0.34 | -0.91 | 0.37 |
| Other Timing/Pausing Measures | | | | | |
| CoVs in Syllable Duration | AMR | -0.74 | 0.48 | -1.55 | 0.12 |
| | SMR | -1.04 | 0.68 | -1.52 | 0.13 |
| | Read | n/a | n/a | n/a | n/a |
| | PB&J | n/a | n/a | n/a | n/a |
| CoVs in Intersyllable Duration | **AMR** | **-0.85** | **0.41** | **-2.04** | **0.04** |
| | SMR | -0.74 | 0.63 | -1.17 | 0.24 |
| | Read | n/a | n/a | n/a | n/a |
| | PB&J | n/a | n/a | n/a | n/a |
| Number of Pauses (per word produced) | AMR | n/a | n/a | n/a | n/a |
| | SMR | n/a | n/a | n/a | n/a |
| | Read | -0.88 | 0.68 | -1.31 | 0.19 |
| | PB&J | -0.34 | 0.5 | -0.68 | 0.49 |

### 2.10.2 Correlations with clinical/motor/risk/variables

As in the main text, we then tested whether these two speech measures were related to symptom severity, non-speech motor measures, as well as risk of conversion variables, as measures of clinical, convergent, and predictive validity. Given that we reported results for Read speech in the main text and its group difference results were just above the significance threshold with the two participants removed, we also report correlation results for Read Speech here.

The consonant results (coefficient of variation of voiceless VOTs) were qualitatively similar to those reported in the main text (Table S3): we found no significant relationships between the speech measure and any of the clinical/motor/risk variables examined in any of the speech samples, with the exception of one significant positive correlation between variability in consonant duration and SIPS-RC risk of conversion scores in the Diadochokinetic-AMR subtask only. The speech rate results were identical to those reported in the main text (Table S4). In particular, as in the main text, we still observed a significant positive relationship between variation in speech rate and variation in finger tapping in the non-dominant hand in the read speech samples.

We observed no other significant correlations between the speech measure and clinical/motor/risk variables. Overall, as in the main text, we generally failed to find evidence of clinical, convergent, and predictive validity of the studied speech measures.

Table S 3: Summary of correlational analyses relating coefficient of variation in voiceless VOTs to symptom, motor, and risk variables with two participants excluded from the analyses.

| Measure | Subtask | Beta | s.e. | z | p |
|---|---|---|---|---|---|
| | | SIPS Symptom Scores | | | |
| Positive Symptoms Total | AMR | 0.45 | 1.96 | 0.23 | 0.82 |
| | SMR | 0.47 | 2.16 | 0.22 | 0.83 |
| | Read | -1.61 | 2.09 | -0.77 | 0.45 |
| Negative Symptoms Total | AMR | 3.96 | 3.12 | 1.27 | 0.21 |
| | SMR | 2.62 | 3.44 | 0.76 | 0.45 |
| | Read | -0.33 | 3.65 | -0.09 | 0.93 |
| Disorganized Symptoms Total | AMR | 0.63 | 1.43 | 0.44 | 0.66 |
| | *SMR* | *2.6* | *1.52* | *1.71* | *0.09* |
| | Read | 1.88 | 1.61 | 1.17 | 0.25 |
| G3: Motor Difficulties | AMR | -0.49 | 0.58 | -0.85 | 0.4 |
| | SMR | 0.61 | 0.63 | 0.97 | 0.34 |
| | Read | -0.43 | 0.69 | -0.62 | 0.54 |
| | | Finger Tapping (Motor) | | | |
| CoV in Finger Tapping (Dominant Hand) | AMR | 0.15 | 0.43 | 0.35 | 0.73 |
| | SMR | 0.28 | 0.47 | 0.59 | 0.56 |
| | Read | 0.38 | 0.47 | 0.8 | 0.43 |
| CoV in Finger Tapping (Non-Dominant Hand) | AMR | 0.02 | 0.39 | 0.06 | 0.95 |
| | SMR | 0.15 | 0.44 | 0.34 | 0.73 |
| | Read | -0.54 | 0.45 | -1.18 | 0.24 |
| | | Risk of Conversion Scores | | | |
| SIPS-RC Risk Score | **AMR** | **2.75** | **1.18** | **2.32** | **0.02** |
| | SMR | 1.91 | 1.35 | 1.41 | 0.16 |
| | Read | 1.02 | 1.47 | 0.7 | 0.49 |

Table S 4: Summary of correlational analyses relating coefficient of variation in speech rates to symptom, motor, and risk variables with two participants excluded from the analyses.

| Measure | Subtask | Beta | s.e. | z | p |
|---|---|---|---|---|---|
| | | SIPS Symptom Scores | | | |
| Positive Symptoms Total | AMR | 1.2 | 0.86 | 1.39 | 0.17 |
| | SMR | -0.25 | 1.02 | -0.25 | 0.81 |
| | Read | 1.02 | 1.74 | 0.59 | 0.56 |
| Negative Symptoms Total | AMR | -0.31 | 1.41 | -0.22 | 0.83 |
| | SMR | -2.45 | 1.59 | -1.54 | 0.13 |
| | Read | -0.73 | 2.93 | -0.25 | 0.8 |
| Disorganized Symptoms Total | *AMR* | *1.19* | *0.66* | *1.81* | *0.08* |
| | SMR | 0.67 | 0.73 | 0.91 | 0.37 |
| | Read | 0.18 | 1.29 | 0.14 | 0.89 |
| G3: Motor Difficulties | AMR | -0.2 | 0.28 | -0.72 | 0.48 |
| | SMR | -0.12 | 0.3 | -0.41 | 0.68 |
| | Read | 0.43 | 0.56 | 0.77 | 0.45 |
| | | Finger Tapping (Motor) | | | |
| CoV in Finger Tapping (Dominant Hand) | AMR | 0.09 | 0.21 | 0.41 | 0.68 |
| | SMR | -0.13 | 0.22 | -0.61 | 0.54 |
| | Read | 0.25 | 0.38 | 0.66 | 0.51 |
| CoV in Finger Tapping (Non-Dominant Hand) | *AMR* | *0.35* | *0.18* | *1.97* | *0.06* |
| | SMR | 0.14 | 0.21 | 0.66 | 0.51 |
| | **Read** | **0.93** | **0.33** | **2.82** | **0.01** |
| | | Risk of Conversion Scores | | | |
| SIPS-RC Risk Score | AMR | 0.28 | 0.61 | 0.45 | 0.65 |
| | SMR | -0.42 | 0.66 | -0.64 | 0.53 |
| | Read | -0.77 | 1.16 | -0.67 | 0.51 |

# 3 Package and environment version

```
## R version 4.1.2 (2021-11-01)
## Platform: aarch64-apple-darwin20 (64-bit)
## Running under: macOS Monterey 12.3.1
##
## Matrix products: default
## BLAS:   /Library/Frameworks/R.framework/Versions/4.1-arm64/Resources/lib/libRblas.0.dylib
## LAPACK: /Library/Frameworks/R.framework/Versions/4.1-arm64/Resources/lib/libRlapack.dylib
##
## locale:
## [1] en_US.UTF-8/en_US.UTF-8/en_US.UTF-8/C/en_US.UTF-8/en_US.UTF-8
##
## attached base packages:
## [1] stats     graphics  grDevices utils     datasets  methods   base
##
## other attached packages:
##  [1] tibble_3.1.8     sjmisc_2.8.9     officer_0.6.2    flextable_0.9.1
##  [5] stringr_1.4.1    kableExtra_1.3.4 pROC_1.18.0      ggpubr_0.4.0
##  [9] dplyr_1.0.10     ggplot2_3.4.0    papaja_0.1.1     tinylabels_0.2.3
##
## loaded via a namespace (and not attached):
##  [1] nlme_3.1-153           fontquiver_0.2.1       insight_0.19.0
##  [4] webshot_0.5.4          httr_1.4.4             tools_4.1.2
##  [7] backports_1.4.1        utf8_1.2.2             R6_2.5.1
## [10] sjlabelled_1.2.0       mgcv_1.8-38            DBI_1.1.2
## [13] colorspace_2.0-3       withr_2.5.0            gridExtra_2.3
## [16] tidyselect_1.2.0       curl_4.3.3             compiler_4.1.2
## [19] textshaping_0.3.6      cli_3.4.1              rvest_1.0.3
## [22] xml2_1.3.3             fontBitstreamVera_0.1.1 labeling_0.4.2
## [25] bayestestR_0.13.0      scales_1.2.1           askpass_1.1
## [28] systemfonts_1.0.4      digest_0.6.30          rmarkdown_2.18
## [31] svglite_2.1.0          gfonts_0.2.0           pkgconfig_2.0.3
## [34] htmltools_0.5.3        highr_0.9              fastmap_1.1.0
## [37] rlang_1.0.6            rstudioapi_0.14        httpcode_0.3.0
## [40] shiny_1.7.3            generics_0.1.3         farver_2.1.1
## [43] jsonlite_1.8.3         zip_2.2.2              car_3.1-1
## [46] magrittr_2.0.3         Matrix_1.3-4           parameters_0.20.2
## [49] Rcpp_1.0.9             munsell_0.5.0          fansi_1.0.3
## [52] abind_1.4-5            gdtools_0.3.3          lifecycle_1.0.3
## [55] stringi_1.7.8          yaml_2.3.6             carData_3.0-5
## [58] plyr_1.8.8             grid_4.1.2             promises_1.2.0.1
## [61] crayon_1.5.2           lattice_0.20-45        splines_4.1.2
## [64] cowplot_1.1.1          knitr_1.41             pillar_1.8.1
## [67] uuid_1.0-3             ggsignif_0.6.3         effectsize_0.8.3
## [70] crul_1.3               glue_1.6.2             evaluate_0.18
## [73] fontLiberation_0.1.0   data.table_1.14.2      vctrs_0.5.1
## [76] httpuv_1.6.6           gtable_0.3.1           openssl_2.0.4
## [79] purrr_0.3.5            tidyr_1.2.1            assertthat_0.2.1
## [82] datawizard_0.6.5       xfun_0.35              mime_0.12
## [85] xtable_1.8-4           broom_1.0.1.9000       rstatix_0.7.0
## [88] later_1.3.0            ragg_1.2.5             viridisLite_0.4.1
## [91] ellipsis_0.3.2
```
